# Schisandrin B Suppresses Pancreatic Ductal Adenocarcinoma Through EGFR/AKT/mTOR Signaling and Mitochondrial Dysfunction

**DOI:** 10.3390/ijms27219996

**Published:** 2026-11-07

**Authors:** Woonghee Lee, Gwonhwa Song, Hyocheol Bae

**Affiliations:** 1 Department of Biotechnology, College of Life Sciences and Biotechnology, Korea University, Seoul 02841, Republic of Korea; cleverwhl@korea.ac.kr; 2 Department of Convergent Biotechnology and Advanced Materials Science, College of Life Sciences, Kyung Hee University, Yongin 17104, Republic of Korea

**Keywords:** pancreatic ductal adenocarcinoma, network pharmacology, mitochondrial dysfunction, schisandrin B, EGFR, MD simulation

## Abstract

Pancreatic ductal adenocarcinoma (PDAC) is a highly lethal cancer with poor prognosis due to chemoresistance and limited treatment options. Schisandrin B (Sch B), a bioactive compound from *Schisandra chinensis*, has demonstrated anticancer effects in various malignancies, but its role in PDAC remains unknown. This study explores the antitumor mechanisms of Sch B using network pharmacology, molecular docking, and in vitro and in vivo experiments, focusing on the EGFR/AKT/mTOR signaling pathway and mitochondrial dysfunction. Network pharmacology identified EGFR, CASP3, BCL2, and HSP90 as key candidate targets of Sch B, and molecular docking and MD simulation were used to explore putative interactions with these proteins. In PDAC cells, treatment Sch B for 48 h reduced cell viability. In addition, Sch B suppressed EGFR/AKT/mTOR signaling and induced mitochondrial dysfunction, characterized by loss of mitochondrial membrane potential, increased mitochondrial and cytosolic calcium levels, and elevated ROS production, accompanied by activation of apoptotic signaling. Co-treatment with gemcitabine demonstrated enhanced effects, elevating apoptosis and oxidative stress. Moreover, Sch B significantly reduced tumor growth in a PANC-1 xenograft model, accompanied by decreased protein expression of EGFR, AKT, mTOR, and HSP90 in tumor tissues. These findings suggest that Sch B exerts antitumor activity associated with modulation of EGFR/AKT/mTOR signaling and mitochondrial dysfunction, supporting further investigation of Sch B as a potential therapeutic candidate for PDAC, particularly in combination with standard chemotherapy.

## 1. Introduction

Pancreatic ductal adenocarcinoma (PDAC) is a devastating neoplastic disease with the lowest 5-year survival rate (only 13%), making it one of the deadliest cancers worldwide [1]. Despite improvement of diagnostic tools and medicinal approaches, the prognosis for PDAC remains grim due to its late-stage presentation, intrinsic resistance to chemotherapy, and the tumor’s highly desmoplastic microenvironment [2,3]. Gemcitabine remains a cornerstone in PDAC therapy owing to its potential to suppress DNA synthesis and hamper cell cycle progression at the G1/S phase [4]. However, its clinical utility is hindered by significant challenges, including drug resistance, systemic toxicity, and suboptimal drug delivery within the dense stromal architecture of PDAC tumors. Combination therapies, such as gemcitabine with nab-paclitaxel, and regimens such as FOLFIRINOX have demonstrated improved efficacy over gemcitabine alone [5,6]. However, gemcitabine-based combinations often yield only modest benefits and are constrained by issues such as drug resistance and limited delivery within the tumor microenvironment [7,8]. Moreover, FOLFIRINOX, while more effective in extending survival, is also accompanied by a high toxicity burden, making it suitable only for a subset of patients who can tolerate its intensive side effect profile [6,9]. Therefore, given the limitations of current treatments, there is a critical demand for innovative treatment strategies that can effectively target the molecular drivers of PDAC while minimizing adverse effects.

The epidermal growth factor receptor (EGFR) pathway has been identified as an essential focus for therapeutic intervention due to its pivotal role in PDAC progression, metastasis, and resistance to therapy [10]. EGFR, frequently overexpressed in PDAC, activates downstream signaling cascades such as the AKT/mTOR pathway, known for promoting cell growth, proliferation, survival, and metabolic adaptation [11,12]. Consistent with the clinical relevance of EGFR, analyses of publicly available PDAC patient datasets indicated that elevated EGFR expression was associated with poorer overall survival and higher expression in tumor tissues compared with non-tumor tissues (Figure S1A,B). Despite its promise, direct targeting of EGFR in PDAC has yielded limited clinical benefits, primarily due to intrinsic and acquired resistance mechanisms, including feedback activation of compensatory pathways. To overcome these challenges, multi-targeted therapeutic approaches are gaining attention.

Systems-level computational strategies such as network pharmacology and molecular dynamics (MD) simulation have emerged as a highly effective approach in bioinformatics for addressing these challenges [13,14]. By integrating drug-associated genes, disease-related targets, and molecular interaction networks, network pharmacology facilitates the identification and prioritization of candidate therapeutic targets and signaling pathways [13,15,16]. This systems-level approach is particularly valuable for complex diseases such as PDAC, where multiple interconnected signaling pathways contribute to tumor progression and treatment responses [17,18]. In particular, molecular modeling techniques, including molecular docking and long-timescale MD simulations, facilitate the identification of key drug-target interactions and provide mechanistic insights into compound behavior under physiological conditions. By integrating these computational frameworks with experimental validation, a more robust and predictive understanding of therapeutic efficacy can be achieved [19,20]. Using this approach, schisandrin B (Sch B), a bioactive natural product derived from *Schisandra chinensis*, has emerged as a promising treatment option for various diseases by exerting versatile pharmacological properties, including anti-inflammatory, antioxidant, and neuroprotective activities [21,22,23]. Previous studies have reported that Sch B enhances panitumumab-induced cytotoxicity in colorectal cancer cells [24], suppresses gastric cancer cell growth [25], and inhibits the proliferation of lung adenocarcinoma cells [26]. Furthermore, Sch B enhances doxorubicin sensitivity in drug-resistant ovarian cancer cells [27]. Among the bioactive lignans derived from *Schisandra chinensis*, Sch B was selected based on its previously reported anticancer activity and potential to enhance the efficacy of conventional chemotherapy. In addition, Sch B has been reported to enhance the efficacy of 5-fluorouracil in gastric cancer models [25], and to inhibit ATR kinase activity involved in the DNA damage response [28]. Although other *Schisandra* lignans exhibit diverse pharmacological activities, the present study focused on Sch B rather than comparing the relative anticancer efficacy of different constituents [29]. Nevertheless, the effects of Sch B on EGFR/AKT/mTOR-associated signaling, mitochondrial dysfunction, and its interaction with gemcitabine in PDAC remain insufficiently characterized.

In this study, we investigated the antitumor effects of Sch B in PDAC, focusing on EGFR/AKT/mTOR-associated signaling and mitochondrial dysfunction. Our findings demonstrated that Sch B inhibited PDAC cell growth, induced apoptosis-associated mitochondrial alterations, and suppressed tumor growth in a PANC-1 xenograft model. Furthermore, Sch B enhanced gemcitabine-associated responses in vitro, supporting its potential for further investigation as a therapeutic candidate for PDAC.

## 2. Results

### 2.1. Network-Based Identification of Candidate Targets Associated with Schisandrin B (Sch B), Gemcitabine, and PDAC

**Figure 1:**
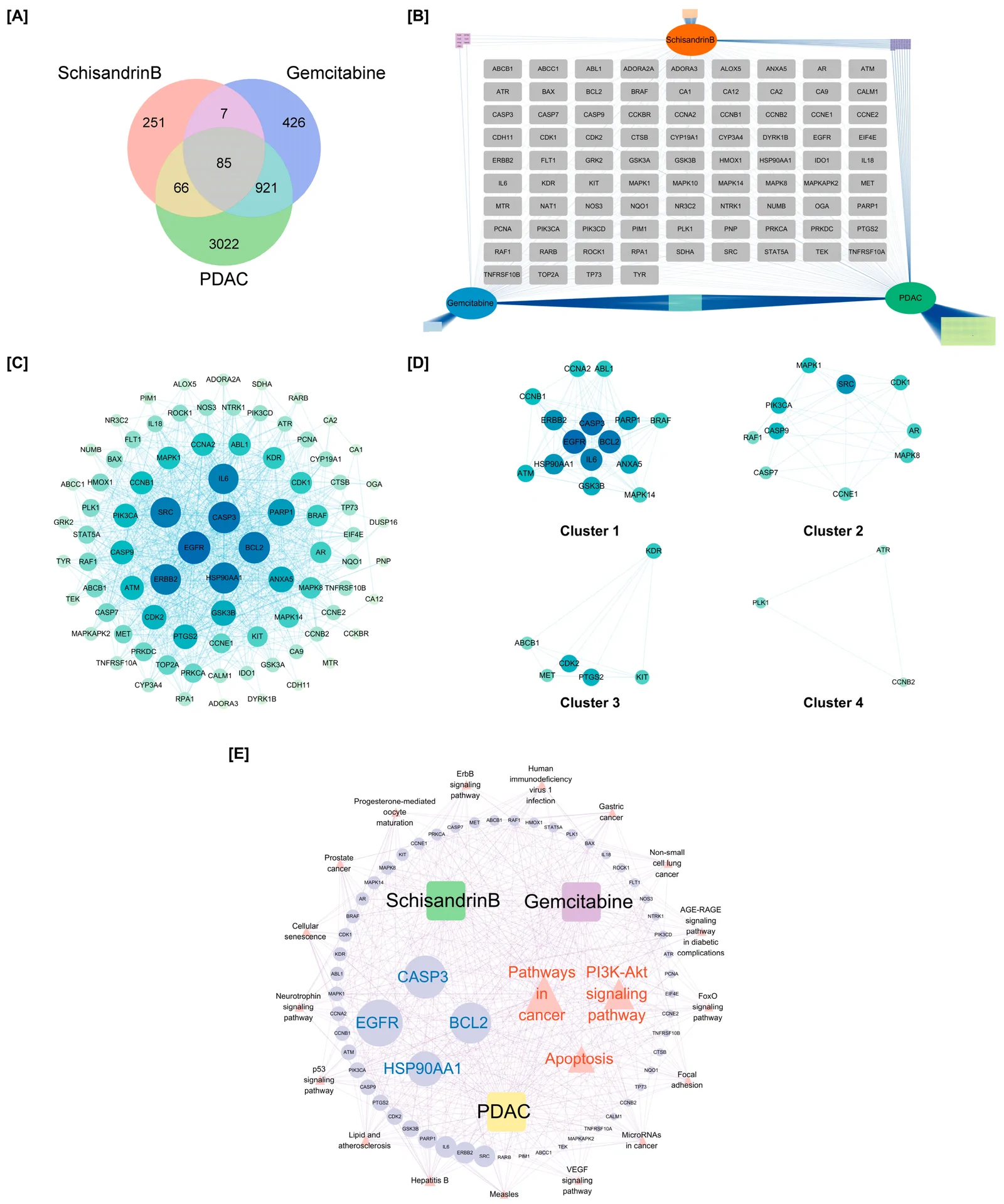
Targets of schisandrin B, gemcitabine, and pancreatic ductal adenocarcinoma (PDAC) identified using network pharmacology. (**A**) Venn diagram illustrating the overlapping and unique targets of schisandrin B, gemcitabine, and PDAC identified from multiple databases. A total of 85 shared targets were identified among the three groups. (**B**) Drug-target-disease network displaying the interaction between schisandrin B, gemcitabine, PDAC, and their associated targets. Common 85 targets were represented in the center (gray). (**C**) Protein–protein interaction (PPI) network explaining connections between 85 shared targets. The size and intensity of the nodes reflect the degree of connectivity, with EGFR, CASP3, BCL2, and HSP90 emerging as central hub targets. (**D**) Functional clusters within the PPI network identified using the MCODE algorithm. (**E**) Drug-Disease-Targets-Pathways network depicting the relationships among schisandrin B, gemcitabine, PDAC, and the key pathways, including cancer pathways, apoptosis, and PI3K-Akt signaling. Nodes represent the targets (blue) and pathways (red), with larger nodes highlighting central targets. Lines indicate the interactions among drugs, targets, and pathways.

To explore the mechanisms by which Sch B and gemcitabine exert their therapeutic impacts on PDAC, a network pharmacology strategy was utilized. Potential targets of Sch B and gemcitabine were retrieved from multiple sources, including GeneCards, HIT, SwissTargetPrediction, STITCH, and PharmMapper. Sch B was found to be associated with a total of 409 identified targets, while gemcitabine was associated with 1006 targets. A total of 4094 targets related to PDAC were obtained from the OMIM, GeneCards, DisGeNET, and TTD databases. After removing duplicates, 85 overlapping targets were identified among Sch B, gemcitabine, and PDAC, as illustrated in the Venn diagram (Figure 1A). A drug-target-disease network was generated and visualized using Cytoscape, illustrating the interactions between Sch B, gemcitabine, and PDAC-related targets (Figure 1B). Moreover, using data from the STRING database, a network illustrating protein–protein interactions (PPI) was developed, employing a medium confidence score of 0.4 (Figure 1C). This network, composed of 85 nodes and 837 edges, demonstrated the interaction strengths between genes, with an average node degree of 19.7. Further network analysis in Cytoscape revealed interaction strength based on node size and color intensity. Core genes, including EGFR, CASP3, BCL2, and HSP90AA1, emerged as key common targets linking Sch B, gemcitabine, and PDAC (Figure 1C). To identify functional clusters within the network, the MCODE algorithm was applied with a node score threshold of 0.1 and a K-core value of 2.0. Four significant clusters were identified (Figure 1D). Cluster 1, with the highest MCODE score (14.286), comprised 15 nodes and 100 edges, highlighting key target genes such as EGFR, BCL2, CASP3, and HSP90AA1. Cluster 2 had a score of 7.111 and included 10 nodes, such as MAPK1, SRC, and CASP9. Cluster 3 consisted of 6 nodes and 13 edges, achieving a score of 5.200 and featuring genes like CDK2, PTGS2, and ABCB1. Cluster 4 consisted of 3 nodes and 3 edges, with an MCODE score of 3.000.

Next, the Enrichr platform was utilized to conduct GO functional and KEGG pathway enrichment analyses for the 85 shared targets of Sch B, gemcitabine, and PDAC. Figure S2A–C present the top 20 enriched categories for biological processes (BP), cellular components (CC), and molecular functions (MF). The biological processes were primarily linked to protein phosphorylation, apoptotic process regulation, and protein modification (Figure S2A). Cellular components were predominantly associated with the cyclin-dependent protein kinase holoenzyme complex, intracellular membrane-bounded organelles, and the nucleus (Figure S2B). Regarding molecular functions, activities such as protein serine/threonine kinase activity, MAPK activity, and protein kinase binding were identified as critical (Figure S2C). The KEGG pathway enrichment analysis revealed several pathways relevant to the targets, with significant pathways including cancer-related pathways, PI3K-Akt signaling, and apoptosis. Figure S2D indicates the top 20 pathways ranked by enrichment scores. Furthermore, a Drug&Disease-Targets-Pathways network was created using Cytoscape by integrating the shared targets, PPI data, and KEGG pathway enrichment results (Figure 1E). Key targets such as EGFR, CASP3, BCL2, and HSP90AA1 were identified as central core nodes due to their strong connectivity and critical roles in apoptosis, PI3K-Akt signaling, and cancer-related pathways. Similarly, pathways such as PI3K-Akt signaling, apoptosis, and those associated with cancer were identified as key contributors to the overall mechanism.

### 2.2. Molecular Docking and MD Simulations of Sch B with Candidate Target Proteins

Molecular docking analysis was performed to explore putative binding poses of Sch B or gemcitabine (GEM) with four candidate proteins prioritized by network pharmacology: EGFR, Caspase-3 (CASP3), HSP90, and BCL-2 (Table 1). The docking scores were determined to be −5.353 kcal/mol for EGFR, −4.128 kcal/mol for CASP3, −6.185 kcal/mol for HSP90, and −4.267 kcal/mol for BCL-2. The interactions among the compounds, proteins, and their structures are shown in Figure S3. To further characterize the predicted binding interactions, comparative docking analyses were performed using representative reference inhibitors of EGFR, CASP3, HSP90, and BCL-2. Erlotinib, Ac-DEVD-CHO, onalespib, and S55746 exhibited docking scores of −8.727, −7.807, −9.095, and −5.288 kcal/mol, respectively (Table S1). The reference inhibitors showed characteristic interactions involving Met769 in EGFR, Arg207 in CASP3, Asp93 and Thr184 in HSP90, and Gln118 and Tyr108 in BCL-2 (Figure S4). Compared with the reference inhibitors, Sch B exhibited distinct predicted interaction patterns, including π-π stacking in EGFR and hydrogen-bond interactions in CASP3, whereas hydrogen-bond interactions were not identified for Sch B in the original HSP90 and BCL-2 docking summaries. These comparisons revealed distinct predicted interaction patterns between Sch B and the reference inhibitors, particularly in hydrogen-bond formation and interacting residues. Although the reference inhibitors generally exhibited more negative docking scores, these differences should not be interpreted as direct measures of relative binding affinity.

**Figure 2:**
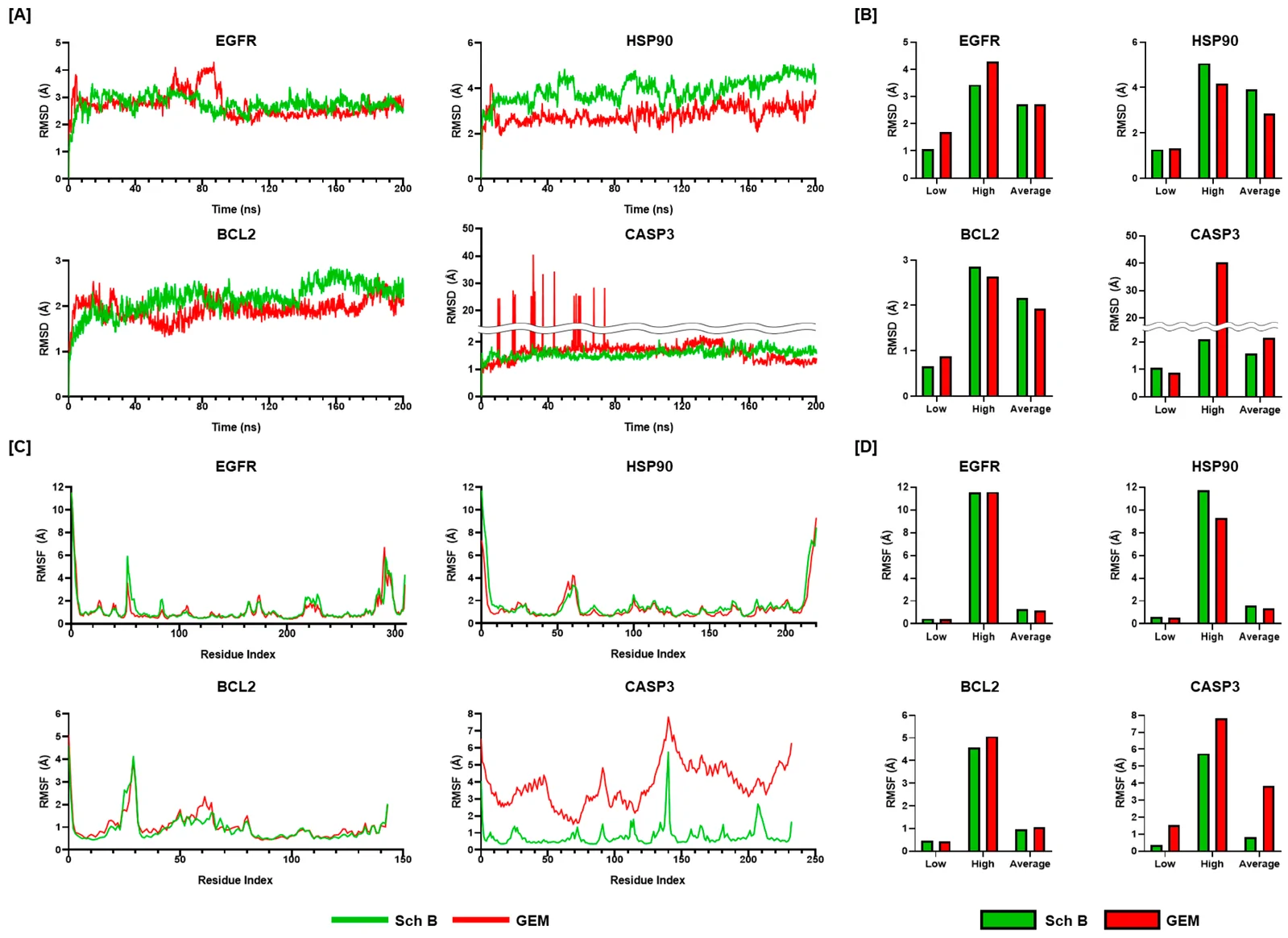
MD simulation results of protein-ligand interactions across a 200 ns timeframe. (**A**) Time-dependent RMSD profiles of the reference ligand gemcitabine (GEM) and schisandrin B (Sch B) bound to EGFR, HSP90, BCL2, and CASP3. (**B**) The lowest, highest, and average RMSD values for GEM and Sch B complexes with the respective target proteins. (**C**) RMSF analysis of each residue for the GEM and Sch B complexes with EGFR, HSP90, BCL2, and CASP3. (**D**) Comparative plot of lowest, highest, and average RMSF fluctuations of the ligands in complex with the four target proteins.

Next, as shown in Figure 2A, RMSDs of Cα atoms were calculated for EGFR, HSP90, BCL2, and CASP3 in complex with Sch B and GEM to assess protein structural stability. In the Sch B-EGFR complex, a relatively stable conformation was observed throughout the 200 ns simulation, with fluctuations largely confined within 1.5–4.0 Å and full stabilization achieved after approximately 40 ns. In contrast, GEM-EGFR exhibited higher deviations with transient peaks reaching 4.286 Å around 85 ns. For HSP90, the Sch B-bound complex exhibited higher RMSD values throughout the 200 ns simulation, fluctuating mostly in the range of 3.0–4.5 Å, with several peaks approaching 5.069 Å. In contrast, the GEM-bound complex maintained more stable conformations, with RMSD values consistently ranging between 2.0 and 3.0 Å, and showing minimal deviation over time. For BCL2, both Sch B- and GEM-bound complexes exhibited relatively stable RMSD profiles after initial equilibration. The Sch B complex stabilized rapidly within the first 20 ns and fluctuated steadily under 2.855 Å throughout the 200 ns simulation. The GEM-bound complex also showed a stable trajectory, with RMSD values maintained under 2.632 Å. For CASP3, the Sch B-bound complex exhibited stable RMSD values throughout the entire 200 ns simulation, with average RMSD exhibiting 1.579 Å. In contrast, the GEM-bound system displayed large and irregular spikes in the RMSD trajectory, particularly during the first half of the simulation (until approximately 80 ns), indicating transient conformational instability or potential local unfolding events. These sharp deviations gradually decreased in the latter half of the simulation but remained above the baseline of the Sch B-bound form. The average RMSD values for Sch B complexes with EGFR (2.713 Å), HSP90 (3.924 Å), BCL2 (2.17 Å), and CASP3 (1.579 Å) were closely aligned with those observed in GEM-bound systems, EGFR (2.723 Å), HSP90 (2.848 Å), BCL2 (1.932 Å), and CASP3 (2.145 Å), indicating that Sch B forms equally stable interactions with these molecular targets (Figure 2B). Regarding residue flexibility, the most pronounced fluctuations in Sch B-bound structures were recorded at Ser52 (5.91 Å) in EGFR, Asp71 (3.364 Å) in HSP90, Glu48 (4.116 Å) in BCL2, and Phe252 (2.688 Å) in CASP3. In comparison, the GEM-bound forms exhibited the highest RMSF at Pro290 (6.706 Å) in EGFR, Asp71 (4.216 Å) in HSP90, Glu48 (3.914 Å) in BCL2, and Glu173 (7.809 Å) in CASP3 (Figure 2C,D). The apo-form RMSD trajectories showed that EGFR, BCL2, and CASP3 reached relatively stable conformational states during the 200 ns simulations, whereas HSP90 displayed comparatively greater structural fluctuations (Figure S5A). The RMSF profiles revealed that the major residue fluctuations were localized mainly to terminal and loop regions rather than reflecting global structural instability (Figure S5B). To further characterize the simulated systems, additional ligand-specific analyses of Sch B were performed, radius of gyration (rGyr), solvent-accessible surface area (SASA), and related molecular surface properties (Figure S6). Furthermore, 200 ns MD simulations of representative reference inhibitor-bound complexes were analyzed using protein and protein-aligned ligand RMSD to provide comparative information on their conformational dynamics (Figure S7). MM-GBSA calculations were also used to provide complementary estimates of binding-related energies for the modeled complexes (Table S1). Altogether, these findings demonstrate that the flexibility profiles of Sch B-bound targets are comparable to those of the reference ligand, supporting the notion that Sch B maintains comparable dynamic stability.

### 2.3. Sch B Lowers PDAC Cell Viability and Triggers Cell Cycle Arrest

**Figure 3:**
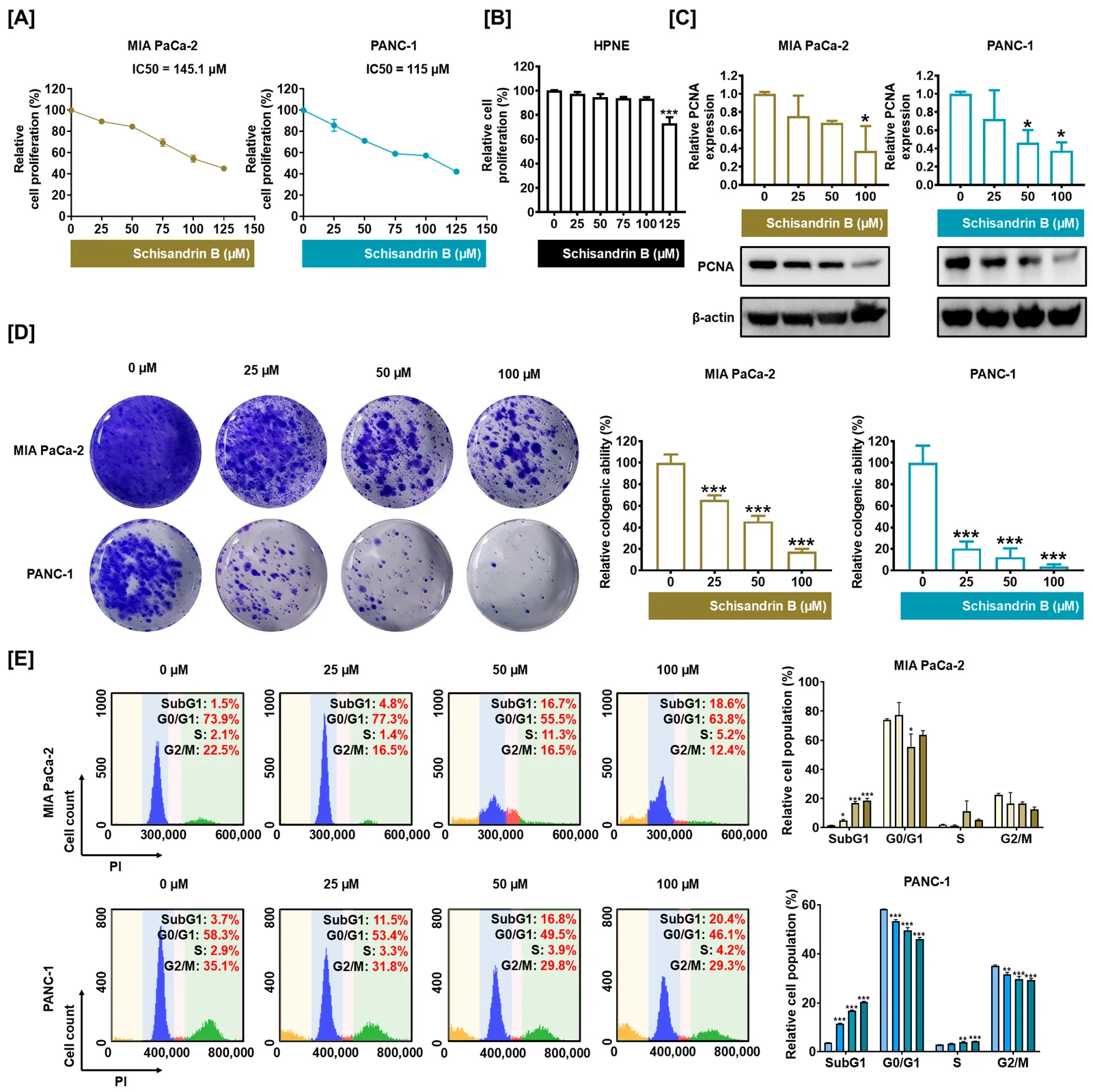
Schisandrin B (Sch B) inhibits pancreatic ductal adenocarcinoma (PDAC) cell proliferation and promotes cell cycle arrest. (**A**,**B**) MTT assay in MIA PaCa-2, PANC-1, and HPNE cells treated with various doses of Sch B for 48 h. Sch B significantly inhibited PDAC cell proliferation gradually while exhibiting no cytotoxicity up to 100 μM in HPNE cells. (**C**) Immunoblotting of PCNA levels in PDAC cells. Sch B treatment resulted in reduced PCNA expression in both cell lines. (**D**) Colony formation assay demonstrates Sch B treatment lowered clonogenic potential of PDAC cells. (**E**) Cell cycle distribution in PDAC cells treated with Sch B. Sch B treatment induced increase in the Sub-G1 population. Triplicate test was performed, * *p* < 0.05, ** *p* < 0.01, and *** *p* < 0.001 denote statistically significant differences compared to untreated controls.

Cytotoxicity of Sch B was evaluated using HPNE and PDAC cells. To assess the potential cytotoxicity of Sch B in non-tumorigenic cells, HPNE cells were included as a comparator in the MTT assay. Sch B exposure exhibited dose-dependent inhibitory impacts on the viability of PDAC cells, whereas it did not exhibit cytotoxicity to HPNE. At concentrations up to 100 µM, PDAC cell viability significantly decreased, with relative proliferation reduced to 54% in MIA PaCa-2 and 57% in PANC-1 at 100 µM. In particular, their IC50 values were 145.1 and 115 µM, respectively (Figure 3A,B). Based on these findings, 100 μM was determined to be the optimal dose for further in vitro experiments. To further evaluate antiproliferative effects of Sch B, protein level of PCNA, a proliferation marker, was assessed by immunoblotting. Sch B treatment significantly reduced PCNA expression with gradual reduction in PDAC cells (Figure 3C). Next, the clonogenic potential of PDAC cells following Sch B treatment was assessed using a colony formation assay. Sch B markedly reduced colony numbers in PDAC cells. At 100 µM of Sch B, colony formation was reduced by 82% and 96%, respectively compared to control (*p* < 0.001), indicating potent inhibition of clonogenic ability (Figure 3D). Additionally, cell cycle alteration induced by Sch B exposure was analyzed using flow cytometry. Sch B exposure markedly elevated Sub-G1 population in PDAC cells, indicating entry of apoptosis (Figure 3E). In MIA PaCa-2, Sub-G1 population rose from 1.5% at 0 µM to 16.7% at 50 µM and 18.6% at 100 µM. Similarly, in PANC-1 cells, the Sub-G1 population increased from 3.7% at 0 µM to 16.8% at 50 µM and 20.4% at 100 µM (*p* < 0.001). Collectively, the above findings demonstrate that Sch B markedly hampers PDAC cell viability and clonogenic potential while inducing cell cycle arrest by increasing Sub-G1 population in PDAC cells.

### 2.4. Sch B Induces Apoptosis and Regulates Apoptosis-Related Protein Expression in PDAC Cells

**Figure 4:**
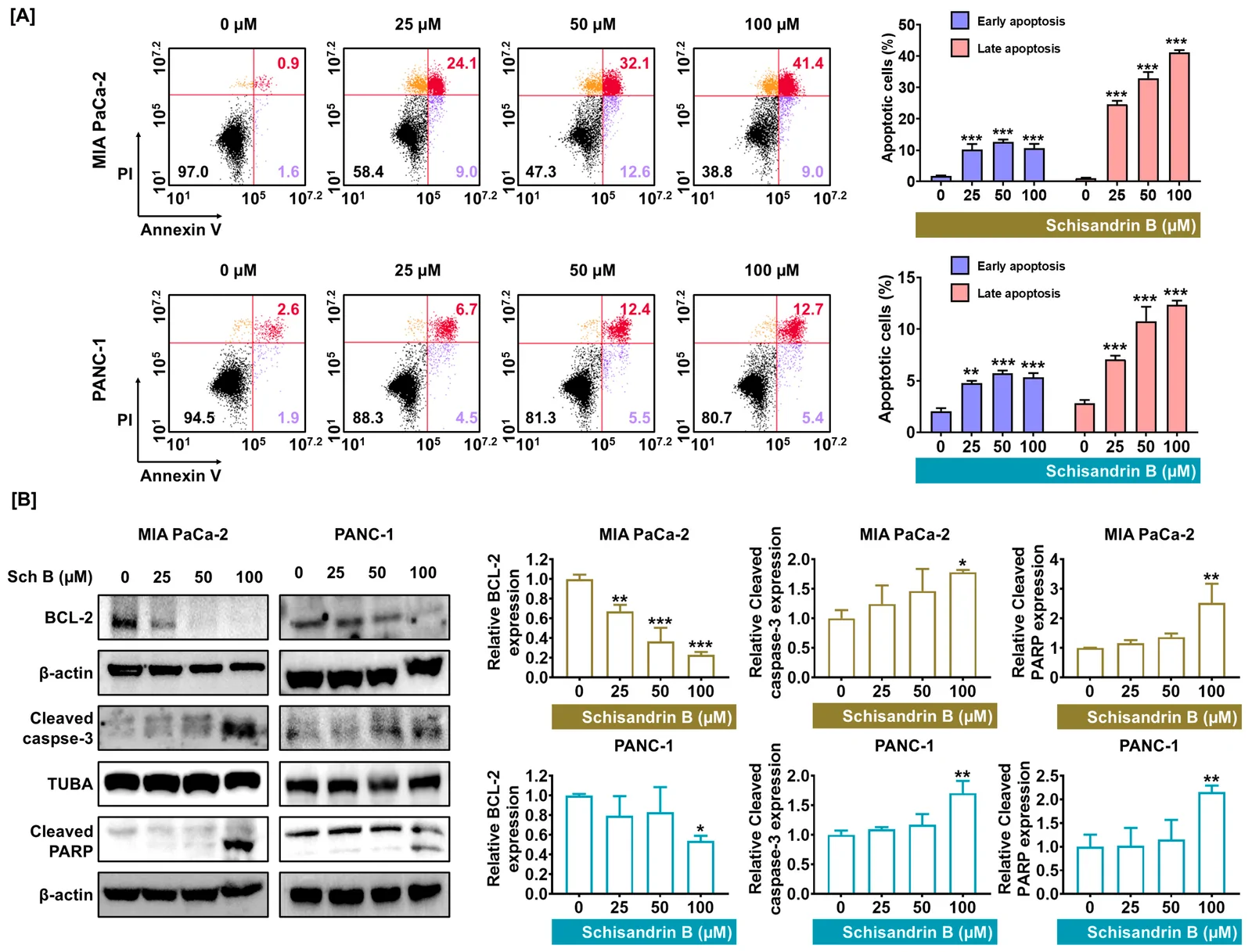
Sch B induces apoptosis and regulates apoptosis-related proteins in PDAC cells. (**A**) Annexin V/PI staining demonstrates apoptosis induction potency of Sch B in PDAC cells. The dot plots indicate early (purple) and late apoptotic (red) cell populations. Sch B exposure significantly increased late apoptotic cells. (**B**) Immunoblotting of apoptosis-related proteins, including BCL-2, cleaved caspase-3, and cleaved PARP. Triplicate test was performed, * *p* < 0.05, ** *p* < 0.01, and *** *p* < 0.001 denote statistically significant differences compared to untreated controls.

Building on prior findings, the pro-apoptotic impacts of Sch B in PDAC were assessed. Annexin V/PI staining proved that Sch B markedly elevated percentage of apoptotic PDAC cells (Figure 4A). Late apoptotic cells in MIA PaCa-2 increased up to 41.4% with 100 μM Sch B treatment, representing an approximately 46-fold increase compared to control group (0 μM). Similarly, late apoptotic cells in PANC-1 increased up to 12.7%, representing an approximately 5-fold increase. The impact of Sch B on apoptosis-related factors was further characterized with immunoblotting investigation (Figure 4B). Sch B significantly downregulated BCL-2 expression in PDAC cells. Relative BCL-2 expression was lowered to 0.23-fold in MIA PaCa-2 and 0.54-fold in PANC-1, respectively, compared to controls group. Moreover, Sch B treatment escalated cleaved caspase-3 and PARP expression, key markers of mitochondrial apoptotic pathways. Cleaved caspase-3 levels increased up to 1.78-fold in MIA PaCa-2 and 1.71-fold in PANC-1 cells, while cleaved PARP expression elevated up to 2.53-fold and 2.16-fold in MIA PaCa-2 and PANC-1, respectively. Therefore, these findings indicate that Sch B triggered apoptosis in PDAC cells by modulating proteins associated with the apoptotic process, implying that Sch B promotes mitochondria-mediated apoptotic pathways, contributing to its anticancer efficacy in PDAC cells.

### 2.5. Sch B Disrupts Mitochondrial Membrane Potential (MMP), Calcium Homeostasis, and Redox Status in PDAC Cells

**Figure 5:**
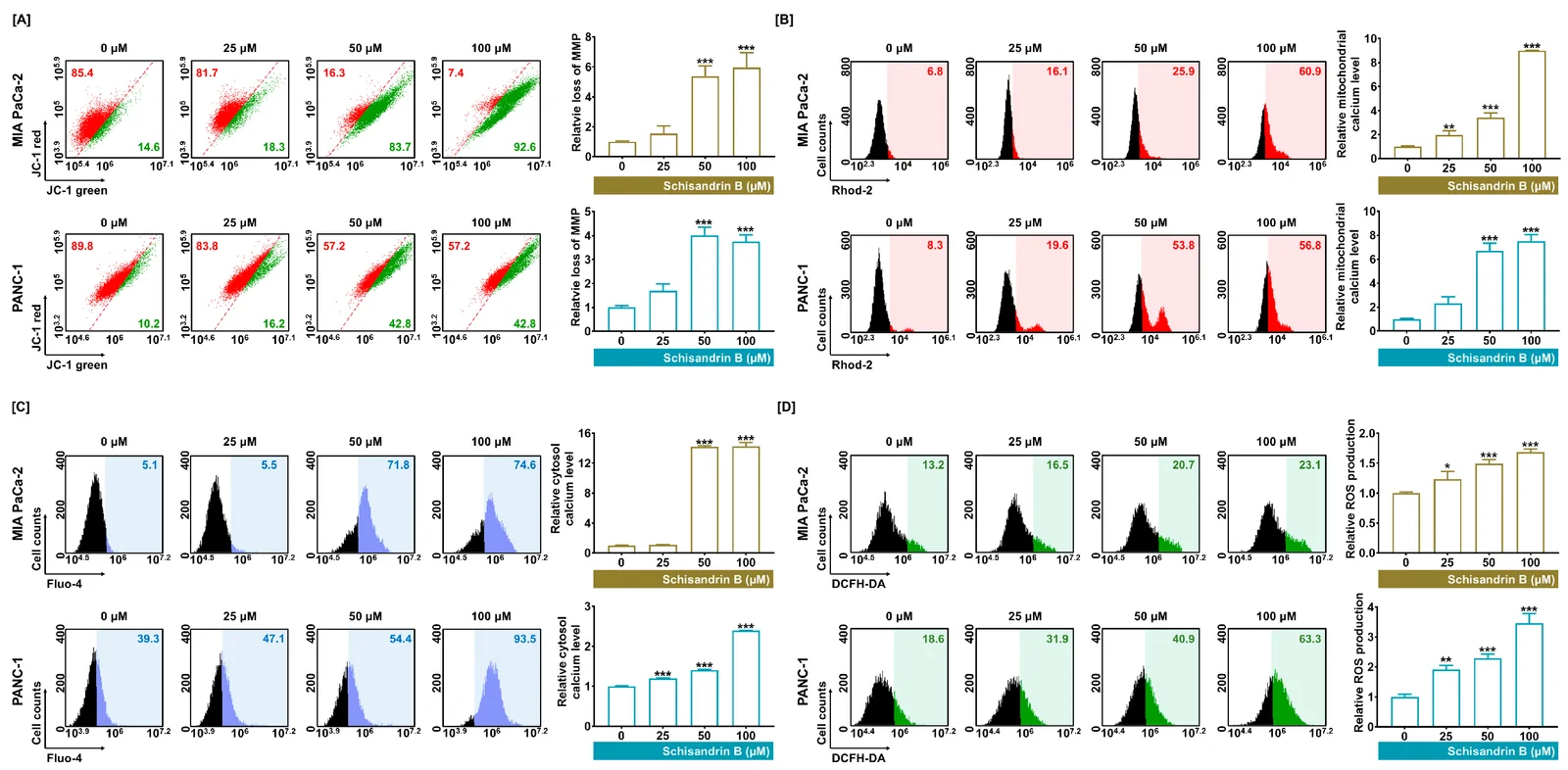
Sch B induces mitochondrial dysfunction and disrupts redox homeostasis in PDAC cells. (**A**) MMP reduction was assessed using JC-1 dye and flow cytometry. Sch B treatment significantly increased the JC-1 monomers, indicating MMP loss. Red and green dots indicate JC-1 aggregates and JC-1 monomers, respectively. (**B**) Sch B markedly elevated mitochondrial calcium levels in both PDAC cells. Red part indicates the Rhod-2 positive cells. (**C**) Sch B significantly increased cytosolic calcium concentrations. Blue part indicates the Fluo-4 positive cells. (**D**) ROS levels were measured using the DCFH-DA fluorescent probe, showing a substantial increase in intracellular ROS production following Sch B treatment. Green part indicates the DCFH-DA positive cells. Triplicate test was performed, * *p* < 0.05, ** *p* < 0.01, and *** *p* < 0.001 denote statistically significant differences compared to untreated controls.

The onset of apoptosis is critically influenced by mitochondrial dysfunction. To investigate whether Sch B induces mitochondrial dysfunction, we assessed the MMP in PDAC cells using JC-1 staining. PDAC cells demonstrated a dose-dependent reduction in MMP upon treatment with Sch B (Figure 5A). At 100 μM, Sch B caused a profound loss of MMP, with reductions reaching 92.6% in MIA PaCa-2 and 42.8% in PANC-1. This depolarization suggests compromised mitochondrial integrity, a hallmark of early apoptotic events. To further evaluate mitochondrial dysregulation, mitochondrial calcium levels were measured using the Rhod-2 fluorescent probe (Figure 5B). Sch B treatment significantly elevated mitochondrial calcium levels. In MIA PaCa-2 cells, relative mitochondrial calcium increased up to 60.9% at 100 μM Sch B, while PANC-1 cells showed a significant rise to 56.8% (both *p* < 0.001). Moreover, we assessed the impact of Sch B on cytosolic calcium levels using Fluo-4 staining dye (Figure 5C). Treatment with Sch B caused a significant rise in cytosolic calcium levels in PDAC. MIA PaCa-2 displayed a 14.2-fold increase at 100 μM, while PANC-1 cells exhibited a 2.4-fold increase (both *p* < 0.001). Next, to explore whether Sch B-induced mitochondrial dysfunction leads to oxidative stress, ROS levels were quantified using the DCFH-DA fluorescent probe (Figure 5D). Sch B treatment significantly elevated intracellular ROS production in PDAC cells. This increase in ROS production suggests that oxidative stress contributes to Sch B-induced apoptotic mechanisms. Collectively, these observations demonstrate that Sch B disrupts MMP, calcium homeostasis, and redox status in PDAC cells, suggesting the role of Sch B in perturbing mitochondrial function and redox homeostasis, ultimately driving apoptosis in PDAC cells.

### 2.6. Sch B Inhibits EGFR/AKT/mTOR Signaling Pathways in PDAC Cells

**Figure 6:**
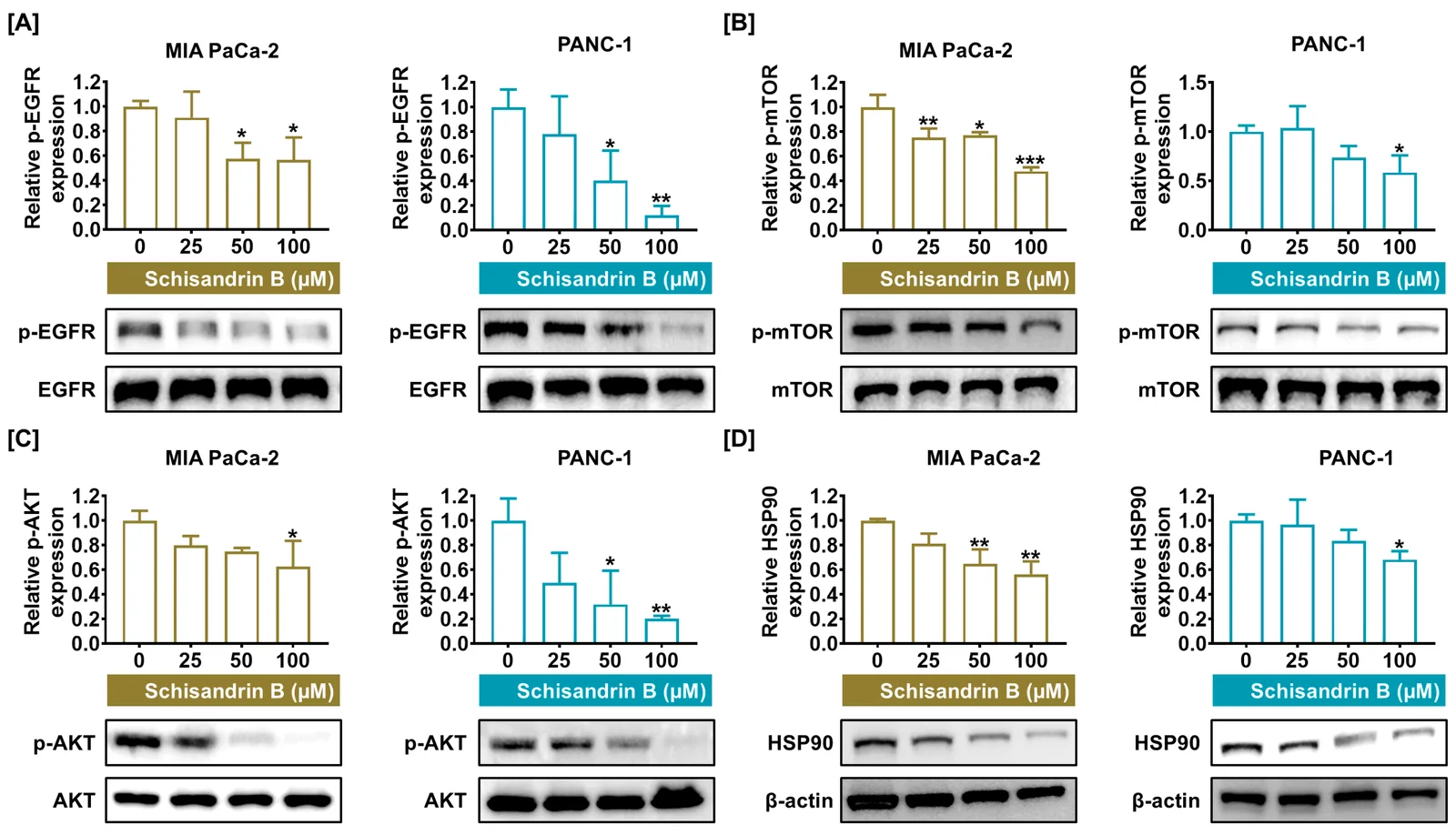
Sch B suppresses EGFR/AKT/mTOR signaling and HSP90 expression in PDAC cells. Panels (**A**–**D**) show the relative protein levels of (**A**) p-EGFR, (**B**) p-mTOR, (**C**) p-AKT, and (**D**) HSP90, normalized against total protein or β-actin. Significance levels of * *p* < 0.05, ** *p* < 0.01, and *** *p* < 0.001, relative to the control group, were determined from experiments conducted in triplicate.

Based on network pharmacology analysis, the EGFR, AKT, and mTOR pathways were identified as key targets of Sch B in PDAC. To validate these findings, immunoblotting was performed to evaluate the impact of Sch B on the protein expression of these targets in PDAC cells. Sch B treatment resulted in a gradual suppression of EGFR phosphorylation in PDAC cells (Figure 6A). At 100 μM, phosphorylated EGFR levels were significantly reduced to 0.57-fold in MIA PaCa-2 and 0.12-fold in PANC-1. In addition, phosphorylated mTOR levels decreased to 0.48-fold in MIA PaCa-2 cells and 0.59-fold in PANC-1 cells following treatment with 100 μM Sch B (Figure 6B). The downstream target AKT, a key protein in the EGFR/mTOR signaling axis, also exhibited significant reductions in phosphorylation upon Sch B treatment (Figure 6C). At 100 μM of Sch B, phosphorylated levels were reduced to 0.63-fold in MIA PaCa-2 and 0.2-fold in PANC-1. HSP90, a molecular chaperone that plays a role in stabilizing and activating various oncogenic factors, has been reported to support the activity of the EGFR/AKT/mTOR signaling axis [30]. HSP90 protein levels decreased to 0.56-fold in MIA PaCa-2 and 0.68-fold in PANC-1 (Figure 6D). These observations imply that Sch B effectively targets and suppresses the EGFR/AKT/mTOR signaling pathways, along with HSP90 downregulation, thereby inhibiting PDAC cell survival and proliferation.

### 2.7. The Knockdown of EGFR Potentiates the Apoptosis and Mitochondrial Dysfunction Induced by Sch B in PDAC Cells

**Figure 7:**
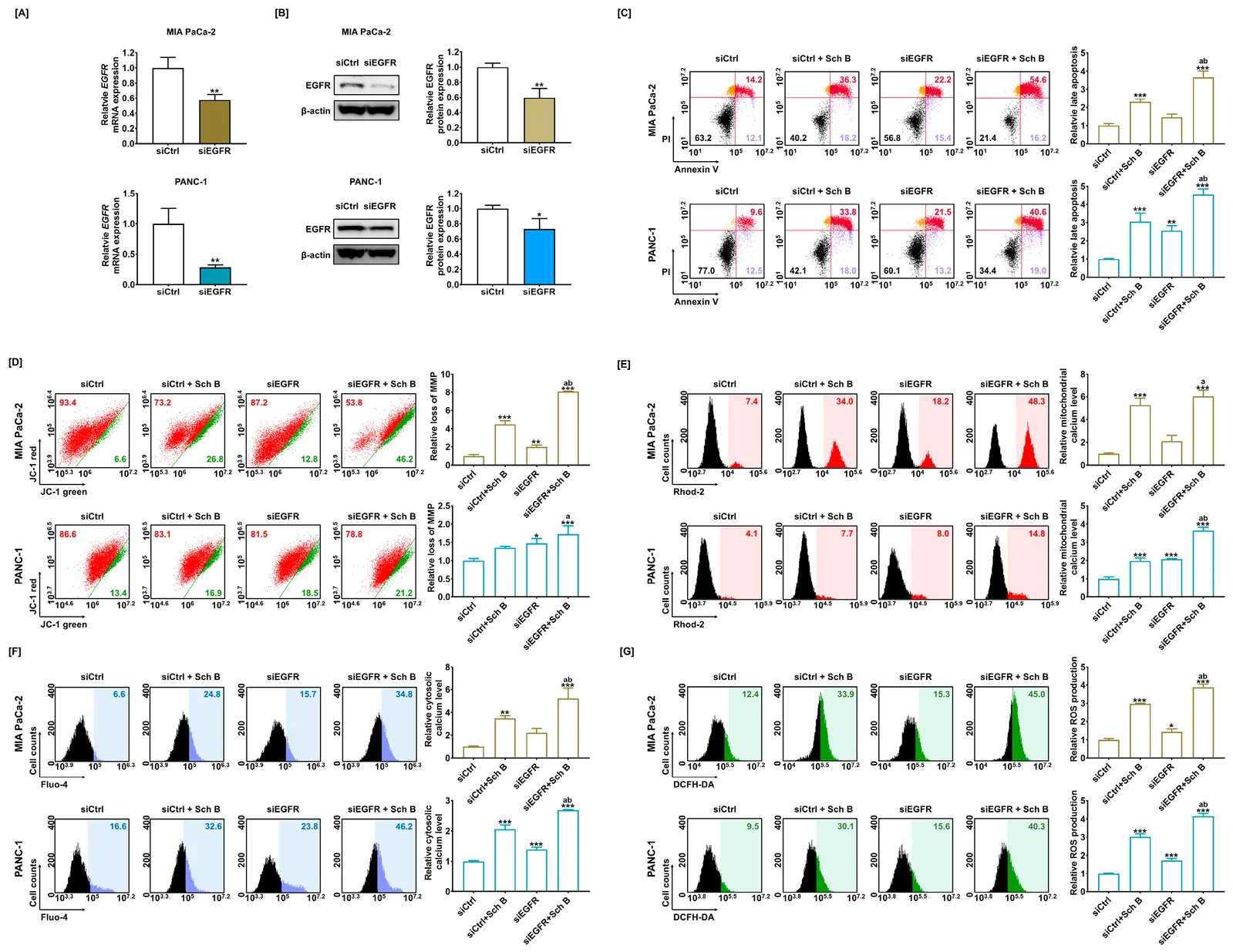
Sch B amplifies apoptosis and mitochondrial dysfunction mediated by EGFR knockdown in PDAC cells. (**A**) qPCR confirmed that EGFR mRNA expression was significantly reduced in PDAC cells treated with siEGFR. (**B**) Western blot analysis showed a pronounced decrease in EGFR protein levels in siEGFR-transfected cells compared to siCtrl. (**C**) Annexin V/PI staining revealed that Sch B treatment significantly increased apoptosis in both siCtrl and siEGFR-transfected cells. Red dots mean late apoptotic cells. (**D**) MMP loss was analyzed using JC-1 staining. Sch B treatment caused a significant increase in MMP loss in siCtrl-transfected cells, with this effect being further amplified by the combination of siEGFR and Sch B. Red and green dots indicate JC-1 aggregates and JC-1 monomers, respectively. (**E**) Mitochondrial calcium levels were evaluated using Rhod-2 dye, showing a significant increase in siEGFR-transfected cells treated with Sch B. Red part indicates the Rhod-2 positive cells. (**F**) Cytosolic calcium levels were assessed with Fluo-4 staining, revealing a substantial elevation in siEGFR-transfected cells treated with Sch B compared to the siCtrl group or individual treatments. Blue part indicates the Fluo-4 positive cells. (**G**) ROS production was measured using DCFH-DA staining, indicating significantly higher ROS levels in cells with EGFR knockdown and Sch B treatment. Green part indicates the DCFH-DA positive cells. Significance levels of * *p* < 0.05, ** *p* < 0.01, and *** *p* < 0.001, relative to the control group, were determined from experiments conducted in triplicate. The symbol “a” indicates a significant effect of Sch B and “b” represents a significant impact of siEGFR.

Accumulating clinical documents suggest that EGFR overexpression is correlated with the progression and poor prognosis of various cancers, including PDAC [31]. To assess the impact of EGFR on the prognosis of patients with PDAC, Kaplan–Meier survival analysis and TNM plot studies were performed [32,33]. Patients with elevated EGFR expression showed significant reductions in overall survival (OS) rates compared to those with lower EGFR levels, as revealed by Kaplan–Meier analysis. (Hazard ratio = 1.6, *p* = 0.031) (Figure S3A). Additionally, analysis of EGFR expression levels in normal (*n* = 108) and tumor (*n* = 248) tissues indicated that EGFR expression was significantly upregulated in PDAC tumors (*p* = 2.15 × 10^−10^) (Figure S3B). These findings suggest the importance of EGFR as an indicator of poor prognosis in PDAC. EGFR knockdown was achieved using siRNA (siEGFR) in PDAC cells to investigate its functional role. The successful knockdown of EGFR expression at both mRNA and protein levels was verified through qPCR and western blot analyses. EGFR mRNA expression was reduced to 0.57-fold in MIA PaCa-2 cells and 0.28-fold in PANC-1 cells (Figure 7A), while protein levels decreased to 0.59-fold and 0.73-fold, respectively (Figure 7B). To investigate the impact of EGFR knockdown on apoptosis, Annexin V/PI staining was performed. Sch B treatment significantly increased late apoptosis in control siRNA (siCtrl)-treated cells, with late apoptotic populations rising to 36.3% in MIA PaCa-2 and 33.8% in PANC-1 cells (Figure 7C). In EGFR-knockdown cells, late apoptosis was further enhanced upon Sch B treatment, reaching 54.6% in MIA PaCa-2 and 40.6% in PANC-1 cells. Notably, Sch B treatment in siEGFR transfected cells exhibited an enhanced effect on apoptosis compared to either siEGFR or Sch B exposure alone.

To further evaluate the function of EGFR in Sch B-induced mitochondrial dysfunction and calcium dysregulation, EGFR was silenced using siEGFR in PDAC cells, and mitochondrial and calcium homeostasis were assessed. While Sch B treatment alone disrupted MMP in siCtrl, with relative MMP loss reaching 26.8% in MIA PaCa-2 and 16.9% in PANC-1, the combination of siEGFR and Sch B further amplified MMP loss, increasing JC-1 green to 46.2% in MIA PaCa-2 and 21.2% in PANC-1 (both *p* < 0.001) (Figure 7D). Additionally, mitochondrial calcium levels were markedly elevated by the combined treatment of siEGFR and Sch B (Figure 7E). Mitochondrial calcium accumulation reached 48.3% in MIA PaCa-2 and 14.8% in PANC-1 cells under combined treatment, representing a significant enhancement compared to either treatment alone (both *p* < 0.001). Similarly, cytosolic calcium levels were evaluated using Fluo-4, revealing an enhanced effect of EGFR knockdown and Sch B treatment (Figure 7F). Cytosolic calcium levels rose to 34.8% in MIA PaCa-2 and 46.2% in PANC-1 cells, significantly higher than levels observed with individual treatments (both *p* < 0.001). Furthermore, Sch B treatment alone elevated ROS production in siCtrl cells, with relative ROS levels reaching 33.9% in MIA PaCa-2 and 30.1% in PANC-1. Notably, the combined treatment of siEGFR and Sch B further increased ROS production to 45.0% in MIA PaCa-2 and 40.3% in PANC-1, demonstrating a significant enhancement compared to individual treatments (both *p* < 0.001) (Figure 7G). Altogether, these findings indicate that EGFR knockdown potentiates Sch B-induced apoptosis, mitochondrial dysfunction, calcium dysregulation, and oxidative stress in PDAC cells, suggesting the therapeutic potential of targeting EGFR in combination with Sch B to enhance its anticancer efficacy.

### 2.8. Sch B Enhances the Efficacy of Gemcitabine in PDAC Cells

**Figure 8:**
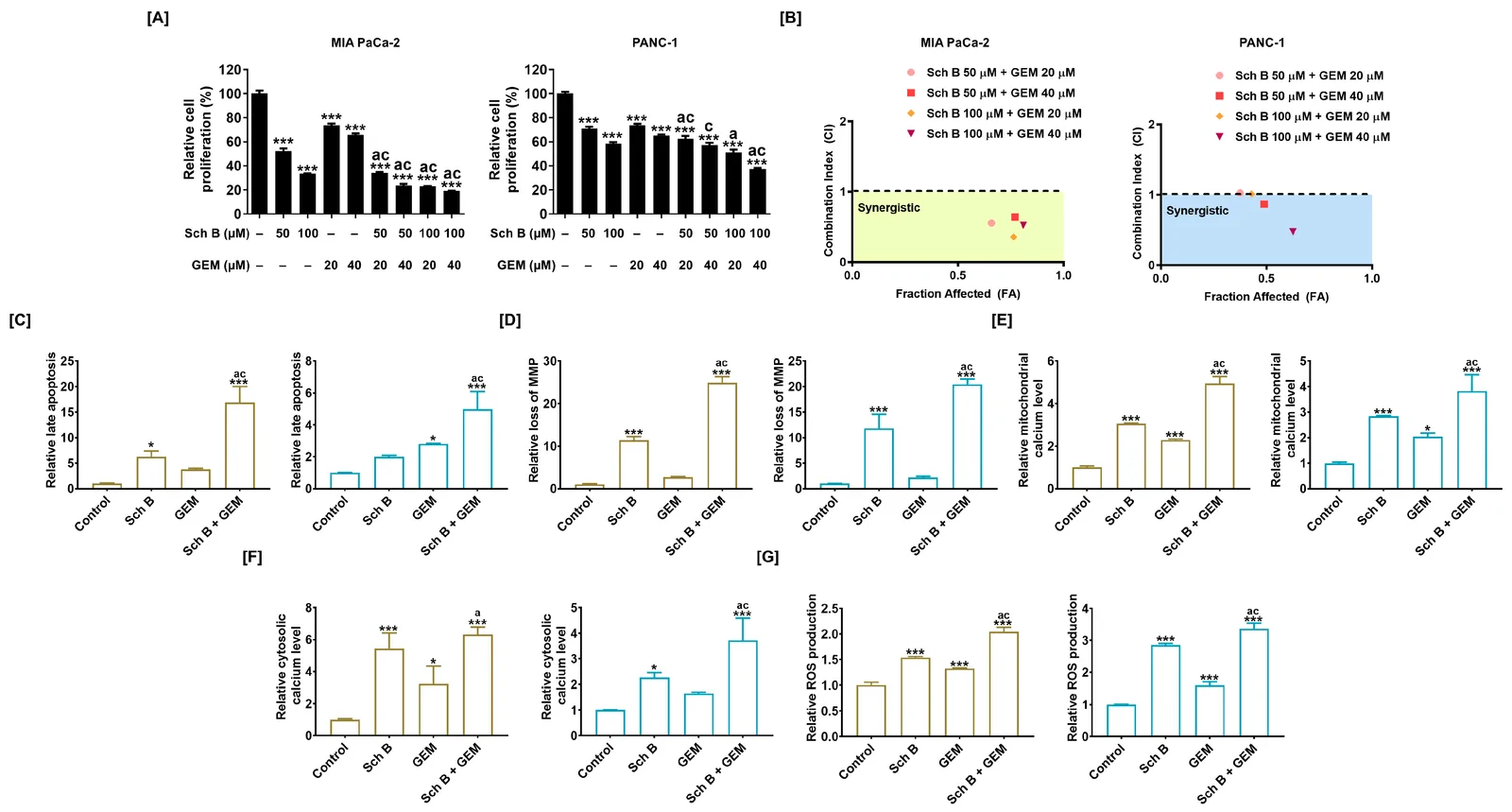
Sch B enhances gemcitabine (GEM) efficacy in PDAC cells. (**A**) Cell proliferation analysis of MIA PaCa-2 and PANC-1 cells treated with various combinations of Sch B and GEM. Combination treatments significantly reduced cell viability compared to single treatments. (**B**) Computational analysis of synergistic effects using the Chou-Talalay method and Compusyn software, demonstrating combination index (CI) values below 1, indicating synergy. (**C**) Late apoptosis was significantly increased by the combination of Sch B and GEM, as measured by Annexin V/PI staining. (**D**) Cotreatment of Sch B and GEM induced enhanced MMP loss, as assessed by JC-1 staining. (**E**) Combination treatments elevated mitochondrial calcium levels measured using Rhod-2 dye. (**F**) Cytosolic calcium levels were significantly increased by cotreatment, as assessed with Fluo-4 staining. (**G**) ROS production was enhanced by the combination treatment, measured using DCFH-DA staining. All experiments were performed in triplicates. * *p* < 0.05, and *** *p* < 0.001 indicate significance compared to the control group. Symbols “a” and “c” represent Sch B- and GEM-induced significance, respectively (*p* < 0.05).

To assess whether Sch B exhibits synergistic effects with gemcitabine (GEM) in PDAC, cell proliferation assays were performed using various drug combinations (Figure 8A). Co-treatment with Sch B (50 or 100 μM) and GEM (20 or 40 μM) significantly reduced cell proliferation in PDAC cells compared to single-drug treatments. Chou-Talalay analysis was performed to evaluate drug interactions using the combination index (CI), where CI < 1, CI = 1, and CI > 1 indicate synergistic, additive, and antagonistic interactions, respectively [34]. Chou-Talalay analysis of the MTT-based proliferation data yielded combination index (CI) values below 1 for all tested combinations in MIA PaCa-2 cells. In PANC-1 cells, CI values below 1 were observed only for combinations containing 40 μM GEM (Figure 8B). These findings indicate that synergy in the proliferation assay depended on the drug concentrations and cell line examined. Further analysis revealed that co-treatment enhanced late apoptosis, depolarization of mitochondrial dysfunction, and oxidative stress compared to individual treatments. Late apoptotic cell populations were significantly higher in co-treated cells, and MMP loss was amplified (Figure 8C,D). Mitochondrial and cytosolic calcium levels were both significantly elevated in the co-treated groups, indicating widespread calcium dysregulation and severe mitochondrial dysfunction (Figure 8E,F). Additionally, ROS production was markedly increased in the co-treatment groups, suggesting enhanced oxidative stress as a critical factor in apoptosis induction (Figure 8G). Collectively, these findings demonstrate that Sch B enhances anticancer effects of GEM, by promoting apoptosis, calcium dysregulation, mitochondrial dysfunction, and oxidative stress, supporting its potential use in combination therapy for PDAC.

### 2.9. Sch B Exhibits Potent Anti-Tumor Effects in a PANC-1 Cell-Derived Xenograft (CDX) Mouse Model

**Figure 9:**
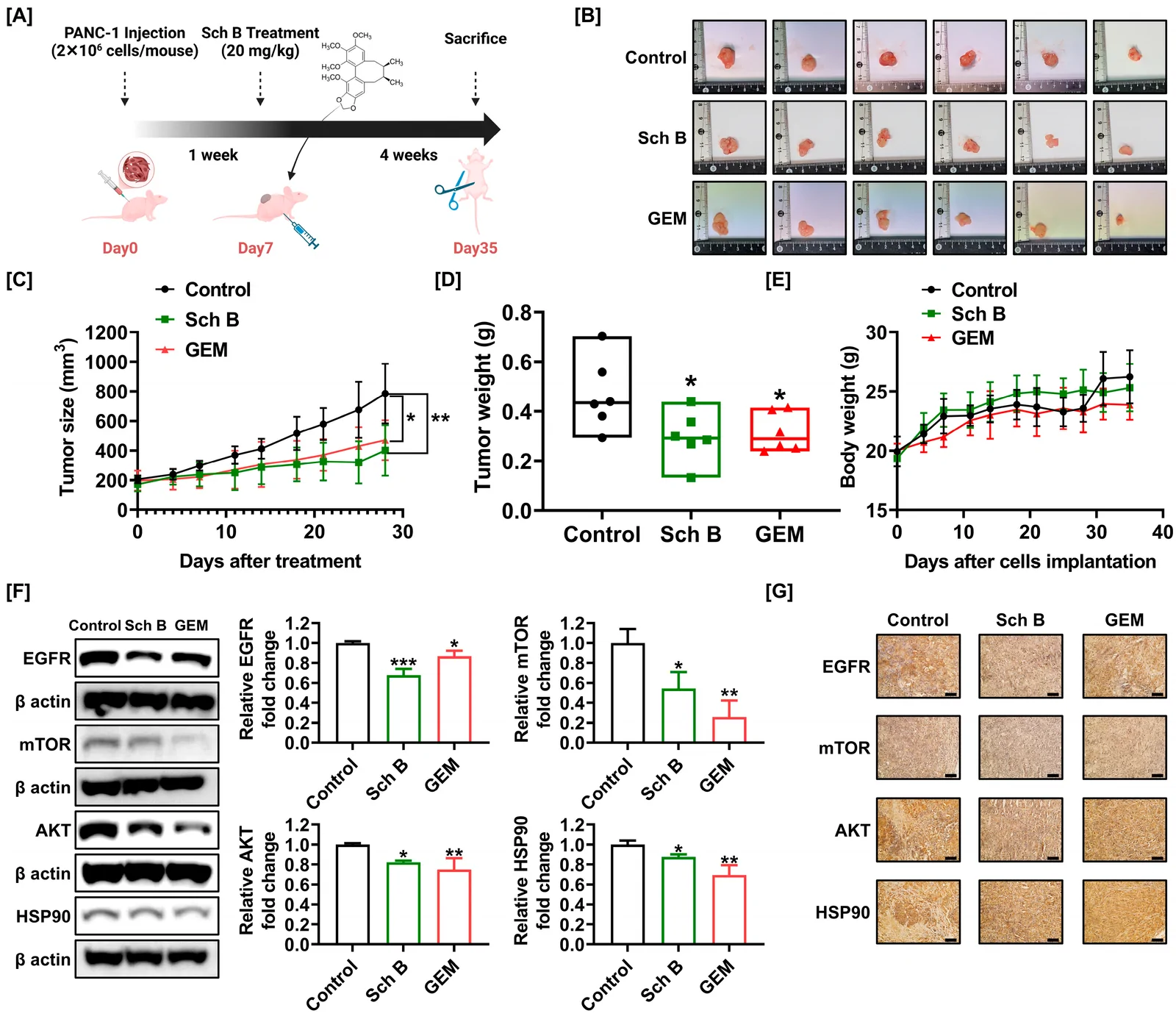
Sch B exhibits potent anti-tumor effects in a PANC-1 cell-derived xenograft (CDX) mouse model. (**A**) Schematic representation of the experimental workflow. PANC-1 cells (2 × 106) were subcutaneously injected into BALB/c nude mice. Mice were treated with vehicle control, Sch B (20 mg/kg), or gemcitabine (GEM; 25 mg/kg) for four weeks. Schematic figure was created in BioRender. Lee, W. (2026) https://BioRender.com/dm8ounx. (**B**) Representative tumor images from different treatment groups at the endpoint. (**C**) Tumor size progression was measured throughout the treatment period, showing significant reductions in the Sch B and GEM groups compared to the control. (**D**) Tumor weights at the endpoint were significantly lower in the Sch B and GEM treatment groups. (**E**) Mouse body weights were monitored throughout the experiment, showing no significant changes across treatment groups (**F**) Western blot analysis of tumor tissues revealed significant downregulation of EGFR, mTOR, AKT, and HSP90 expression in the Sch B-treated group. (**G**) Immunohistochemistry (IHC) confirmed the reduced expression of EGFR, mTOR, AKT, and HSP90 in tumor sections from Sch B and GEM-treated mice. Scale bars represent 100 µm. Significance levels of * *p* < 0.05, ** *p* < 0.01, and *** *p* < 0.001 indicate statistically significant differences relative to the control group. N = 6 mice per group, with each mouse considered an independent biological replicate.

To evaluate in vivo antitumor potency of Sch B, PANC-1 CDX mouse model was established. Mice were injected with either Sch B (20 mg/kg) or gemcitabine (GEM, 25 mg/kg) as a positive control (Figure 9A). Sch B treatment significantly inhibited tumor progression compared to the control group (Figure 9B). Tumor size analysis revealed that Sch B effectively reduced tumor growth, showing a more noticeable decline in tumor size, comparing GEM at the end of the treatment period (Figure 9C). Tumor weights were also significantly lowered in both the Sch B-treated and GEM-treated groups compared to the control, further supporting the potent antitumor potency of Sch B (Figure 9D). Notably, no noticeable changes in body weight were observed among the treatment groups during the experimental period (Figure 9E). In addition, as consistent with in vitro findings, western blot analysis of tumor tissues exhibited that Sch B treatment effectively suppressed protein level of EGFR, mTOR, AKT, and HSP90, which are key signaling proteins implicated in PDAC progression (Figure 9F). Furthermore, IHC staining confirmed the inhibition of these targets in tumor tissues, with markedly lower expression of EGFR, mTOR, AKT, and HSP90 in the Sch B-treated group compared to the control (Figure 9G). Therefore, these findings suggest that Sch B exhibits significant anti-tumor activity in vivo, by targeting the EGFR/AKT/mTOR signaling pathway and suppressing HSP90 expression.

## 3. Discussion

PDAC is a devastating neoplastic disease, characterized by a 5-year survival rate of 13% [1]. This poor prognosis is attributed to late-stage diagnoses, intrinsic resistance to chemotherapy, and a dense desmoplastic microenvironment that hinders effective drug delivery [35,36]. Although gemcitabine remains a cornerstone in PDAC treatment, its clinical utility is significantly limited by challenges such as drug resistance and systemic toxicity. Moreover, FOLFIRINOX, while offering improved survival, is often unsuitable for many patients due to its high toxicity profile [6]. These limitations suggest an urgent need for novel therapies that target the molecular drivers of PDAC while minimizing adverse effects. Recent studies highlight the potential of natural products in drug development, with approximately 51% of new small-molecule drugs approved globally between 1981 and 2014, including around half of all anti-tumor drugs, derived directly from plants or modified semi-synthetically [37]. Therefore, natural compounds are widely recognized as valuable sources for developing new drug candidates, particularly for adjuvant or supportive cancer treatments, including PDAC, owing to their diverse pharmacological properties and potential cost-effectiveness [38,39]. In this regard, we identified schisandrin B (Sch B), a bioactive compound derived from Schisandra chinensis, as a potent therapeutic agent for PDAC. Our findings demonstrate that Sch B inhibits PDAC progression through multiple mechanisms, including EGFR/AKT/mTOR axis inhibition, mitochondrial dysfunction induction, and enhancement of gemcitabine efficacy. These results imply that Sch B may address the unmet clinical needs in PDAC therapy.

Sch B has shown remarkable anticancer effects in various malignancies, including lung, brain, and gallbladder cancer cells. Lv et al. have reported that Sch B suppressed proliferation by reducing PCNA expression and inducing cell cycle arrest in lung adenocarcinoma cells [26]. Evidence has suggested that Sch B significantly suppress cell proliferation of malignant glioma cells, as assessed by colony formation and MTT assay [40]. Similarly, in nasopharyngeal carcinoma, Sch B promotes cell cycle arrest at the G1 phase by modulating the level of cyclins and cyclin-dependent kinases [41]. Consistent with these findings, our study revealed that Sch B exerts similar anticancer effects in PDAC. in vitro experiments showed a reduction in PCNA expression, an increase in the Sub-G1 population indicative of apoptosis, and significant suppression of clonogenic ability in PDAC cells. In addition, the in vivo efficacy of Sch B was evaluated in a PANC-1 cell-derived xenograft mouse model, where Sch B treatment significantly suppressed tumor growth without causing significant changes in body weight during the experimental period. The dose of Sch B (20 mg/kg) used in our study was selected based on previous studies [42,43]. Tumor growth inhibition appeared more pronounced in the Sch B-treated group than in the gemcitabine-treated group under the experimental conditions tested, suggesting potential antitumor activity of Sch B in this model. Moreover, Western blot and IHC analyses revealed reduced protein levels of EGFR, AKT, mTOR, and HSP90 in tumor tissues. These findings are consistent with the reduced EGFR/AKT/mTOR phosphorylation and HSP90 expression observed in vitro, where Sch B also induced mitochondrial dysfunction and apoptosis. Although these results suggest an association between alterations in EGFR/AKT/mTOR-related signaling and tumor growth inhibition, the underlying mechanisms remain to be further validated in vivo.

The in vivo experiments were primarily designed to evaluate the antitumor efficacy of Sch B monotherapy, with gemcitabine serving as a positive control, rather than to assess combination efficacy. Therefore, the therapeutic effects of Sch B combined with gemcitabine remain to be validated in vivo. Although no significant changes in body weight were observed, this finding alone does not exclude potential systemic toxicity. Further hematological, biochemical, and histopathological evaluations are needed to comprehensively assess the safety profile of Sch B. The subcutaneous xenograft model was selected for an initial assessment of the antitumor effects of Sch B because it allows convenient and reproducible monitoring of tumor growth and treatment responses. However, this model does not fully recapitulate the pancreatic tumor microenvironment, particularly the stromal architecture and tumor-host interactions, which may influence therapeutic responses [44,45]. Therefore, further validation using orthotopic PDAC models is warranted to evaluate the antitumor effects of Sch B in a more physiologically relevant setting. Altogether, these results suggest the chemotherapeutic potency of Sch B in PDAC, providing a strong preclinical foundation for its further development against PDAC.

The use of network pharmacology represents a paradigm shift in drug discovery, particularly for complex diseases like PDAC, where single-target therapies often fail due to compensatory pathway activation and drug resistance [13,15,46]. Traditional approaches to drug discovery are linear, focusing on single molecules or pathways, which limits their applicability in diseases involving interconnected molecular networks. In contrast, network pharmacology enables the identification of multitarget compounds and elucidates their mechanisms of action through disease-gene-target-drug networks [15]. In this study, network pharmacology analysis identified EGFR, CASP3, BCL2, and HSP90 as candidate targets of Sch B in PDAC. From a structural perspective, EGFR contains an intracellular ATP-binding kinase domain targeted by inhibitors such as erlotinib, whereas HSP90 possesses an N-terminal ATP-binding pocket targeted by inhibitors such as onalespib (AT13387) [47,48]. BCL-2 contains a hydrophobic BH3-binding groove targeted by selective inhibitors such as S55746, while CASP3 is an executioner cysteine protease with a catalytic substrate-binding site inhibited by peptide-based compounds such as Ac-DEVD-CHO [49,50]. EGFR, in particular, plays a pivotal role in PDAC progression by regulating the AKT/mTOR axis, involved in cell growth, survival, and metabolic adaptation [11]. Several studies have highlighted the clinical significance of EGFR, showing its overexpression or abnormal activation in various solid tumors, including PDAC, and linking its activity to patient prognosis [51,52]. Previous studies have shown that erlotinib, an EGFR inhibitor, or combination with gemcitabine can improve survival in PDAC patients, but their clinical benefits are often modest due to resistance mechanisms [53,54]. In our study, Sch B effectively downregulated EGFR/AKT/mTOR signaling pathway. Furthermore, Sch B downregulated HSP90, a chaperone protein that stabilizes oncogenic proteins, including those involved in the EGFR axis [55]. Molecular docking and MD simulations were used to explore putative interactions between Sch B and EGFR, HSP90, BCL-2, and CASP3. Molecular docking provided predicted binding poses and scores, whereas MD simulations characterized the structural dynamics of the modeled protein-ligand complexes. Although relatively pronounced RMSD fluctuations were observed in the HSP90 complexes, such deviations may reflect intrinsic conformational flexibility, as previously reported for the HSP90 N-terminal domain [56]. Furthermore, protein Cα RMSD primarily reflects conformational changes and does not independently establish the stability or dissociation of bound ligands. These computational findings complement the network-based prioritization of candidate proteins but do not establish direct physical binding or cellular target engagement. Similarly, the observed changes in protein abundance, phosphorylation, and caspase-3 cleavage reflect treatment-associated cellular responses rather than evidence that Sch B directly binds these proteins. Experimental binding and cellular target-engagement assays were not performed in the present study, and the proposed Sch B-protein interactions therefore remain unverified. Furthermore, docking scores and RMSD/RMSF measurements cannot be directly used to determine reliable dissociation constants (Kd), as they do not provide sufficiently accurate estimates of standard binding free energies [57,58]. Therefore, Kd values were not derived from these computational results. Accordingly, these proteins should be considered candidate targets for further investigation rather than experimentally validated direct molecular targets.

Mitochondrial dysfunction is a hallmark of cancer cell apoptosis and a promising therapeutic target in PDAC. Growing research indicates that targeting mitochondrial function offers a promising approach for cancer therapy by influencing calcium levels, membrane potential, and oxidative stress regulation [59,60]. The mitochondrial, or intrinsic, apoptosis pathway is initiated by perturbations in mitochondrial homeostasis, including the loss of MMP and release of cytochrome c into cytosol. This triggers caspase activation, ultimately driving cell death. Evidence has shown that calcium dysregulation, through elevated mitochondrial and cytosolic calcium levels, contributes to mitochondrial membrane permeabilization and triggers apoptotic pathways in cancer cells [61]. ROS further amplifies these effects by causing oxidative damage and destabilizing mitochondrial integrity, exacerbating apoptosis [62,63]. In our study, Sch B induced significant dose-dependent reductions in MMP, elevated mitochondrial and cytosolic calcium levels, and increased ROS production in PDAC cells, collectively indicating mitochondrial dysfunction. These mitochondrial perturbations disrupted redox homeostasis and activated key players in the intrinsic apoptosis pathway. Additionally, Western blot analysis revealed a substantial downregulation of the anti-apoptotic protein BCL-2, which is crucial for maintaining mitochondrial integrity by preventing the release of cytochrome c [64]. Moreover, Sch B treatment exhibited significant elevation of cleaved PARP and cleaved caspase-3 expression, markers of apoptosis execution. Cleaved caspase-3 acts as a critical effector caspase, while PARP cleavage is a hallmark of DNA damage-induced apoptosis. Cleaved caspase-3 and PARP cleavage are widely used as markers of apoptosis, supporting the ability of Sch B to induce apoptosis through the mitochondrial pathway [65,66]. Our findings provide insight into the role of EGFR in regulating mitochondrial activity, redox balance, and apoptotic mechanisms in PDAC. Importantly, EGFR knockdown enhanced Sch B-induced mitochondrial dysfunction, calcium dysregulation, and oxidative stress, suggesting that EGFR signaling may modulate the mitochondrial response to Sch B. Therefore, these findings suggest that Sch B targets mitochondrial dysfunction, at least in part, through regulation of the EGFR/AKT/mTOR signaling pathway.

Combination therapy represents an important strategy for improving therapeutic efficacy in PDAC by targeting complementary cellular processes [67]. For example, Hong et al. reported that liriopesides B enhanced gemcitabine-induced apoptosis in pancreatic cancer cells through suppression of AKT/NF-κB signaling and BCL-2/survivin expression [68], consistent with our findings of reduced AKT phosphorylation and BCL-2 expression following Sch B treatment. In contrast, Wei et al. demonstrated that gemcitabine combined with cisplatin induced ferroptosis through the Sp1-SAT1-polyamine metabolism pathway in PDAC [69]. Furthermore, a previous study has reported heterogeneous responses to Metavert combined with conventional chemotherapeutic agents in patient-derived PDAC organoids, suggesting the importance of molecular subtype and treatment history [70]. Similarly, Cen et al. demonstrated that cathayanon E enhanced oxaliplatin sensitivity by promoting MCL1 degradation and mitochondrial apoptosis in colorectal cancer, including in vivo combination effects [71]. Collectively, these studies imply distinct molecular strategies for enhancing chemotherapy responses. In our study, Chou-Talalay analysis demonstrated concentration- and cell-line-dependent synergy between Sch B and gemcitabine. Co-treatment also enhanced apoptosis, mitochondrial dysfunction, calcium dysregulation, and ROS production, although these endpoints were not formally evaluated for synergy. These findings suggest that Sch B may enhance gemcitabine responses through apoptosis-associated and mitochondrial changes. Although FOLFIRINOX is an established multidrug chemotherapy regimen for PDAC, gemcitabine was selected as a well-established chemotherapeutic agent because the present study specifically aimed to evaluate its interaction with Sch B [9]. Therefore, direct comparisons between Sch B and FOLFIRINOX were beyond the scope of this study and warrant further investigation.

## 4. Materials and Methods

### 4.1. Drug and Disease Target Prediction

The prospective human targets of schisandrin B (Sch B) and gemcitabine were predicted through several web-based platforms, including GeneCards, HIT, STITCH, SwissTargetPrediction, and PharmMapper. Following retrieval, target identifiers were converted to gene names via the UniProt ID mapping service, and redundant entries were excluded to yield a non-overlapping gene set. Similarly, human genes associated with PDAC were collected from GeneCards, DisGeNET, TTD, and OMIM databases. After deduplication, a consolidated list of disease-related genes was assembled. Subsequently, the target profiles of schisandrin B, gemcitabine, and PDAC were input into the bioinformatics platform to identify common targets using a Venn diagram. Shared genes were further visualized using Cytoscape (v3.10.3) to construct an interaction network.

### 4.2. Protein–Protein Interaction (PPI) Network Analysis

PPI data were collected using the STRING database (https://string-db.org/) (accessed on 27 November 2025) with a minimum interaction confidence score of 0.400. The PPI network was generated using Cytoscape software, where node properties such as color, size, and width were continuously mapped according to the degree layout. To group the targets based on their specific biological functions, the MCODE plug-in in Cytoscape was used, applying a node score cutoff of 0.1 and a K-Core value of 2.0. Relevant targets associated with schisandrin B, gemcitabine, and PDAC, along with PPI and KEGG pathway data, were imported into Cytoscape to build a ‘Drug&Disease-Targets-Pathways’ network. Only key targets and pathways were selected and visualized in the final network.

### 4.3. GO and KEGG Pathway Analysis

Functional enrichment analyses were conducted using the Enrichr tool to explore Gene Ontology (GO) categories, covering biological processes (BP), cellular components (CC), and molecular functions (MF), as well as KEGG pathways. The top 20 significantly enriched terms were subsequently illustrated using the bioinformatics online visualization platform.

### 4.4. Molecular Docking

The interaction of Sch B with EGFR, HSP90, BCL-2, and Caspase-3 was evaluated through molecular docking using the Maestro software package (Schrödinger, New York, NY, USA), following a previously described protocol [72]. Briefly, the 2D structure of Sch B was retrieved in SDF format from the PubChem database (https://pubchem.ncbi.nlm.nih.gov/) (accessed on 27 November 2025) [73]. The three-dimensional crystal structures of EGFR (PDB ID: 6LUD), HSP90 (PDB ID: 2XJX), BCL-2 (PDB ID: 6GL8), and Caspase-3 (PDB ID: 3DEI) were obtained from the RCSB Protein Data Bank [74]. These experimentally determined human protein structures were selected to provide structural templates for docking analysis based on their available crystallographic coordinates and relevant protein domains. Detailed structural information, including UniProt identifiers, mutation status, resolution, experimental method, and release dates, is provided in Table S1. The original co-crystallized ligands and other non-protein components were removed, and the retained protein structures were prepared using Maestro’s Protein Preparation Wizard. Sch B was processed separately using LigPrep. Potential binding sites were identified using SiteMap, and docking was performed in standard precision (SP) mode using the Ligand Docking Wizard. This procedure followed the same protocol as described in our previous study [42]. For comparative docking analysis, representative inhibitors of EGFR (erlotinib), CASP3 (Ac-DEVD-CHO), HSP90 (onalespib), and BCL-2 (S55746) were prepared and docked using the same computational workflow. The resulting docking poses were analyzed to identify interacting amino acid residues and hydrogen-bond interactions for comparison with Sch B.

### 4.5. Molecular Dynamic (MD) Simulation

MD simulations were performed for EGFR, HSP90, BCL-2, and Caspase-3 complexed with either schisandrin B or gemcitabine at their optimal docking conformations, utilizing the Desmond package in Maestro (Schrödinger, New York, NY, USA). Each protein-ligand system was prepared with the OPLS4 force field and solvated in a TIP3P water model within a minimized orthorhombic simulation box, maintaining a 10 Å padding. To ensure system neutrality, ions were adjusted with a 0.15 M salt concentration. Prior to production runs, all systems underwent energy minimization and equilibration under standard settings. Simulations were then conducted over 200 ns in the NPT ensemble at 300 K and 1.013 bar. Post-simulation, trajectory data were analyzed using the Simulation Interaction Diagram tool to assess interaction dynamics, with RMSD and RMSF values extracted for structural stability and flexibility evaluations. To provide ligand-free control references for comparison with the ligand-bound systems, additional MD simulations were performed for the apo forms of EGFR, HSP90, BCL2, and CASP3. For each apo system, the co-crystallized or docked ligand was removed, and the protein was simulated using the same preparation, equilibration, and 200 ns production run protocol as used for the ligand-bound complexes. MM-GBSA calculations were used to estimate binding-related energies, with the resulting values summarized in the corresponding tables.

### 4.6. Bioinformatic Analysis

The expression patterns of EGFR mRNA in both healthy and PDAC tissues were examined using the TNMplot resource (https://tnmplot.com/analysis/) (accessed on 15 November 2025) [33], incorporating data from 356 non-tumor and pediatric tissue samples. Furthermore, the prognostic impact of EGFR expression on overall survival (OS) was assessed in 178 PDAC cases via the GEPIA platform (http://gepia.cancer-pku.cn/) (accessed on 15 November 2025), which provided automated calculations for Log-rank *p*-values and hazard ratios (HRs).

### 4.7. Reagents

Schisandrin B (99.94% purity) and gemcitabine were sourced from Medchem Express (Monmouth Junction, NJ, USA) and prepared in dimethyl sulfoxide (DMSO). Details of the antibodies employed are outlined in Table 2.

### 4.8. Culture and Treatment Conditions

MIA PaCa-2 and PANC-1 pancreatic cancer cell lines (passage numbers <30), commonly used in PDAC studies, were acquired from the Korean Cell Line Bank (Seoul, Republic of Korea), while the HPNE non-tumoral pancreatic ductal cell line (passage numbers <10) was obtained from ATCC (Molsheim, France). PDAC cells were grown in DMEM supplemented with fetal bovine serum and antibiotics, whereas HPNE cells were cultured following ATCC guidelines. Cells were seeded into 60 mm dishes or multi-well plates (6- or 96-well formats) and cultured until reaching ~70% confluence. Following overnight serum deprivation, cells were treated with the indicated concentrations of Sch B.

### 4.9. Cell Proliferation Assay

To evaluate Sch B’s impact on cell growth in PDAC and HPNE lines, the Cell Proliferation Kit (Roche, Basel, Switzerland) was utilized. Cells were treated with different concentrations of Sch B for 48 h, followed by the addition of MTT reagent. After incubation at 37 °C for 4 h, a solubilization buffer was added. The absorbance of the formed purple formazan was recorded using a microplate reader at dual wavelengths of 595 nm and 620 nm. IC50 value was calculated by simple nonlinear regression using GraphPad Prism 9.0.2 (La Jolla, CA, USA).

### 4.10. Determination of Protein Expression with Western Blots

Proteins were isolated from PDAC cells, and the expression levels of target proteins were analyzed through western blotting, following the methodology described in our previous study [75]. Protein quantification was carried out using β-actin or α-tubulin (TUBA) as loading controls, depending on the molecular weight of the proteins.

### 4.11. Colony Formation Analysis

For the colony formation assay, PDAC cells were seeded in 6-well plates at a density of 2000 cells per well and treated with different concentrations of Sch B for 24 h, following the method outlined in a prior study [72]. Colonies consisting of over 50 cells were observed using microscopic analysis and their relative clonogenic potential was quantified using ImageJ 1.53e software. Each experimental setup was performed in triplicate.

### 4.12. Measurement of the Mitochondrial Membrane Potential (MMP)

The mitochondrial membrane depolarization was assessed using a mitochondrial staining kit (Sigma-Aldrich, St.Louis, MO, USA). PDAC cells were treated with Sch B at concentrations of 0, 25, 50, and 100 μM for 48 h. After treatment, the cells were collected and stained with JC-1 dye for 20 min. Changes in MMP were analyzed using flow cytometry.

### 4.13. Detection of Late Apoptotic Cell

Apoptosis in PDAC cells treated with Sch B was verified using the apoptosis detection kit I (BD Biosciences, Franklin Lakes, NJ, USA), according to the protocol detailed in an earlier study [76].

### 4.14. Cell Cycle Analysis

The effect of Sch B on cell cycle distribution in PDAC cells was examined using flow cytometry, adhering to the methods outlined in a previous study [77].

### 4.15. Assessment of Mitochondrial and Cytosolic Calcium Ion

Calcium levels in the mitochondria and cytosol of PDAC cells were assessed using Rhod-2 and Fluo-4 dyes, respectively (Invitrogen, Carlsbad, CA, USA). PDAC cells were exposed to different concentrations of Sch B for 48 h. Following treatment, the cells were stained with either Rhod-2 or Fluo-4 dyes, washed thoroughly, and the fluorescence intensity was measured using flow cytometry.

### 4.16. Reactive Oxygen Species (ROS) Assay

PDAC cells were plated in 6-well plates and exposed to Sch B at concentrations of 0, 25, 50, and 100 µM or a combination of Sch B with gemcitabine in a medium containing 2% FBS for 1 h. Following this treatment, the cells were incubated with DCFH-DA dye (Sigma-Aldrich) for 30 min and subsequently washed with PBS. The fluorescence intensity of DCF, formed through the interaction of DCFH-DA with ROS, was measured using a flow cytometer.

### 4.17. Quantitative Real-Time PCR

Total RNA was extracted using the TransZol Up reagent (TransGen Biotech, Beijing, China) according to the manufacturer’s protocol. cDNA was synthesized with AccuPower^®^ RT PreMix (Bioneer, Daejeon, Republic of Korea), and quantitative PCR was conducted using SYBR Green chemistry on the CFX Connect Real-Time PCR System (Bio-Rad, Hercules, CA, USA), following the methodology reported by a previous study [78]. EGFR-specific primers were designed using Primer3 software with the following sequences: forward 5′-TAA CAA GCT CAC GCA GTT GG-3′ and reverse 5′-AGG GCA ATG AGG ACA TAA CC-3′.

### 4.18. Gene Silencing by siRNA Transfection

EGFR expression was downregulated by transfecting PDAC cells with siEGFR using Lipofectamine 2000 (Invitrogen, Carlsbad, CA, USA), as per the manufacturer’s instructions (Bioneer, Daejeon, Republic of Korea). The siEGFR duplex included the sense strand 5′-GAU CCA CAG GAA CUG GAU ATT-3′ and the antisense strand 5′-UAU CCA GUU CCU GUG GAU CTT-3′. Both siEGFR (final concentration: 50 nM) and a scrambled control siRNA (siCtrl) were purchased from Bioneer’s predesigned genome-wide siRNA collection. For transfection, Lipofectamine and siRNA were each diluted in Opti-MEM (Gibco-BRL, Waltham, MA, USA) and allowed to sit for 5 min at room temperature before being mixed and incubated for 20 min to allow complex formation. The resulting mixture was applied to PDAC cells for 48 h, followed by PBS washing and subsequent Sch B or vehicle treatment.

### 4.19. Determination of Synergistic Effect

The interaction between schisandrin B and gemcitabine was assessed through the Chou-Talalay approach [34], which quantifies drug synergy. Based on proliferation assay data, varying concentrations and response levels were selected using a non-fixed ratio design to evaluate combined treatment effects. Optimal dosing conditions were established empirically, and the combination effects were analyzed computationally to generate median-effect curves. According to the Chou-Talalay method, CI values < 1, =1, and >1 indicate synergistic, additive, and antagonistic interactions, respectively.

### 4.20. Animal Experiments and Xenograft Models

To investigate the therapeutic efficacy of schisandrin B in PDAC, male BALB/c nude mice (4 weeks old, ~20 g) were obtained from Orient Bio (Seongnam, Republic of Korea) and maintained under specific pathogen-free conditions. The animals were housed at 23 ± 1 °C with regulated humidity and a 12 h light/dark cycle, and provided ad libitum access to food and water. After a one-week acclimatization period, a cell line-derived xenograft (CDX) model was generated by subcutaneously injecting PANC-1 cells (2 × 106 cells/mouse) mixed 1:1 with PBS and Matrigel (BD Biosciences) into the flanks. Tumor volume was recorded biweekly using the formula V = 0.5 × length × width2. When tumors reached approximately 200 mm^3^, the mice were randomly assigned to three groups (*n* = 6 per group; each mouse was considered an independent biological replicate) and treated intraperitoneally with vehicle (10% DMSO in PBS), Sch B (20 mg/kg, five times weekly), or gemcitabine (25 mg/kg, twice weekly). After 28 days, animals were euthanized, and tumors were excised for weighing and further analysis. All procedures were approved by the Kyung Hee University Institutional Animal Care and Use Committee (IACUC) (approval no. KHGASP-24-530,approval date. 7 October 2024) and adhered to the International Council for Laboratory Animal Science standards and the ARRIVE guidelines [79]. Group allocation was randomized, and outcome assessments were performed in a blinded manner to ensure objectivity.

### 4.21. Immunohistochemistry (IHC)

To evaluate the expression patterns of EGFR, AKT, mTOR, and HSP90 in tumor tissues, immunohistochemical staining was performed using the Vectastain ABC Kit (Vector Laboratories, CA, USA). Tumor specimens were embedded in paraffin, sliced into sections, deparaffinized with xylene, and sequentially rehydrated using decreasing concentrations of ethanol (100% to 70%). Antigen retrieval was achieved by heating the slides in citrate buffer, after which endogenous peroxidase activity was quenched using 3.3% hydrogen peroxide. The sections were then blocked and incubated overnight at 4 °C with primary antibodies against the target proteins (1:200 dilution). After washing, an HRP-linked secondary antibody was applied at 37 °C for 1 h. Visualization was accomplished using a DAB substrate solution, followed by counterstaining with Mayer’s hematoxylin for 15 s. The slides were then rinsed, dehydrated, and coverslipped. Stained tissue morphology was observed microscopically.

### 4.22. Statistics

Prior to statistical testing, data distribution was assessed using the Shapiro–Wilk normality test. For triplicate experiments with homogenous variances, comparisons between two groups were conducted using unpaired *t*-tests, while multiple group comparisons were assessed via one-way ANOVA followed by Tukey’s multiple comparisons test. In cases where variances were unequal, Brown–Forsythe and Welch ANOVA were applied, with Dunnett’s T3 post hoc analysis performed subsequently. All statistical procedures were executed using GraphPad Prism version 9.0.2. Data are presented as mean ± standard deviation (SD), and statistical significance was defined as a *p*-value < 0.05.

## 5. Conclusions

In conclusion, this study presents a comprehensive computational and experimental framework demonstrating that Sch B exerts robust anticancer activity against PDAC. Through a systems-level network pharmacology approach, EGFR, CASP3, BCL2, and HSP90 were identified as key molecular targets, which was validated by molecular docking MD simulations, revealing stable binding interactions. in vitro analyses confirmed that Sch B suppresses EGFR/AKT/mTOR signaling and induces mitochondria-mediated apoptosis through disruption of membrane potential, calcium homeostasis, and redox balance. Notably, EGFR knockdown further amplified these effects, underscoring the regulatory role of EGFR in mitochondrial integrity. Sch B also exhibited synergistic efficacy in vitro with gemcitabine, enhancing apoptosis and oxidative stress. Moreover, Sch B significantly reduced tumor growth in a xenograft mouse model, further corroborating its therapeutic potential. Collectively, this integrative study highlights Sch B as a promising multitarget agent that leverages network-based target identification and dynamic simulation validation to overcome current limitations in PDAC therapy. Several limitations should be acknowledged. Although our findings demonstrate the antitumor effects of Sch B, the proposed molecular targets require direct experimental validation, and the clinical relevance of the concentrations used in vitro remains uncertain. Furthermore, the absence of in vivo combination-treatment experiments, limited toxicity assessments, and use of a subcutaneous xenograft model warrant further investigation to establish the therapeutic potential of Sch B in PDAC. Nevertheless, our findings support further translational development of Sch B as a candidate for monotherapy or combination therapy in clinical oncology.

## Figures and Tables

**Table 1:** The detailed molecular docking results.

Target Proteins	Protein PDB ID	Docking Score (kcal/mol)		MM-GBSA (kcal/mol)		Interactions	
		Schisandrin B	Gemcitabine	Schisandrin B	Gemcitabine	Schisandrin B	Gemcitabine
EGFR	6LUD	−5.353	−5.03	−41.87	−29.72	π-π stacking	Hydrogen bonds
CASP3	3DEI	−4.128	−5.136	−21.39	−21.43	Hydrogen bonds	Hydrogen bonds
HSP90	2XJX	−6.185	−6.79	−41.28	−19.02	-	Hydrogen bonds
BCL-2	6GL8	−4.267	−5.23	−38.04	−24.58	-	Hydrogen bonds

**Table 2:** The detailed information about antibodies we used.

Antibody	Supplier	Use	Dilution	Catalog Number
PCNA	Proteintech	Western blot	1:1000	10205-2-AP
BCL-2	Proteintech	Western blot	1:1000	12789-1-AP
Caspase-3	Proteintech	Western blot	1:1000	19677-1-AP
p-AKT	Cell signaling Technology	Western blot	1:1000	4060
p-mTOR	Cell signaling Technology	Western blot	1:1000	5536
p-EGFR	Cell signaling Technology	Western blot	1:1000	3777
AKT	Cell signaling Technology	Western blot	1:1000	9272
		IHC	1:200	
mTOR	Cell signaling Technology	Western blot	1:1000	2983
		IHC	1:200	
EGFR	Cell signaling Technology	Western blot	1:1000	4267
		IHC	1:200	
HSP90	Cell signaling Technology	Western blot	1:1000	4877
PARP	Cell signaling Technology	Western blot	1:1000	9542
β-Actin	Santa Cruz Biotechnology	Western blot	1:1000	sc-47778
α Tubulin	Santa Cruz Biotechnology	Western blot	1:1000	sc-32293

## Data Availability

All datasets presented in this study can be obtained by contacting the corresponding author.

## Supplementary Materials

The following supporting information can be downloaded at https://www.mdpi.com/article/10.3390/ijms27219996/s1.
