# Supplementary material for: Schisandrin B Suppresses Pancreatic Ductal Adenocarcinoma Through EGFR/AKT/mTOR Signaling and Mitochondrial Dysfunction
Source: Int J Mol Sci. 2026 Nov 7;27(21):9996. doi: 10.3390/ijms27219996 (PMC13764619; doi:10.3390/ijms27219996)
Supplement: Supplementary file 1 [file ijms-27-09996-s001.zip › ijms-4628420-supplementary.pdf]

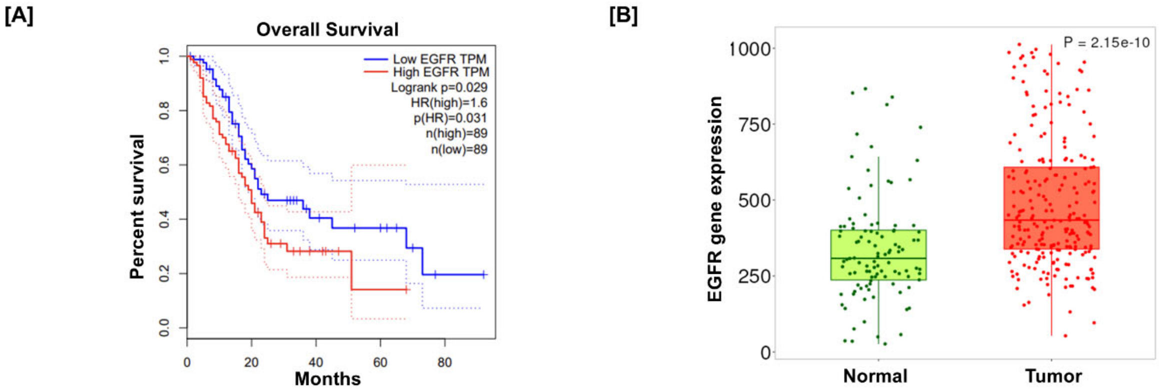

Figure S1. Clinical relevance of EGFR expression in pancreatic ductal adenocarcinoma (PDAC). [A] Kaplan-Meier survival analysis of PDAC patients indicates that elevated EGFR expression is linked to worse overall survival (Hazard ratio = 1.6, p = 0.031). [B] TNMplot analysis comparing EGFR gene expression between normal (n = 108) and tumor (n = 248) tissues reveals significantly higher EGFR levels in PDAC tumors (p = 2.15e-10).

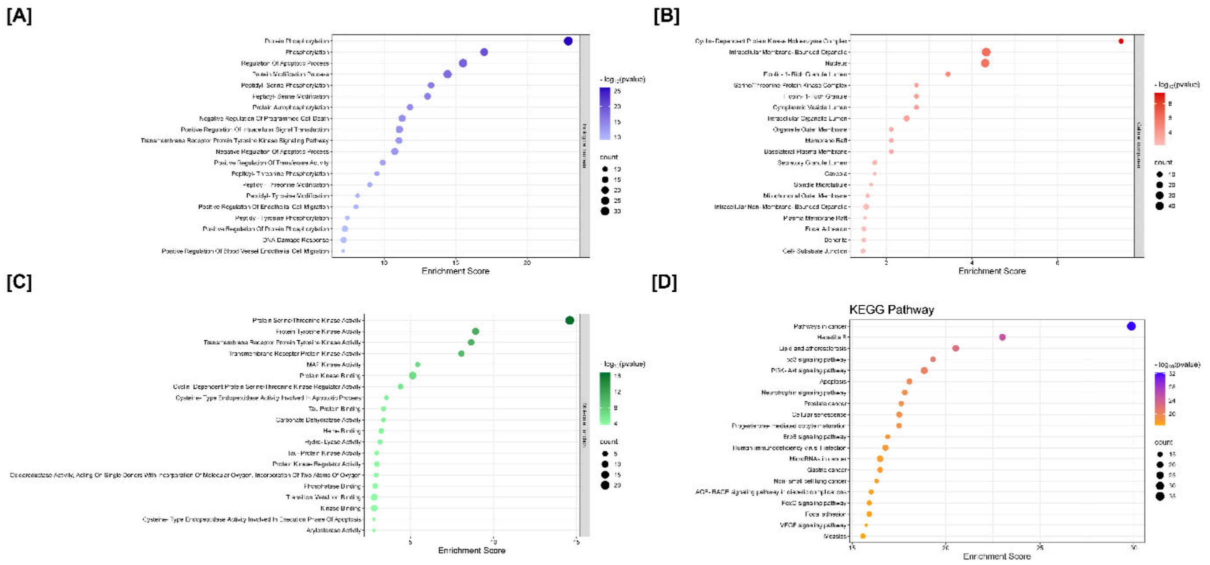

Figure S2. GO functional and KEGG pathway enrichment analyses for the shared targets of Sch B, gemcitabine, and PDAC. [A-C] The major 20 enriched [A] biological process (BP), [B] cellular component (CC), and [C] molecular function (MF) terms identified through GO analysis. [D] KEGG pathway enrichment analysis of the shared targets.

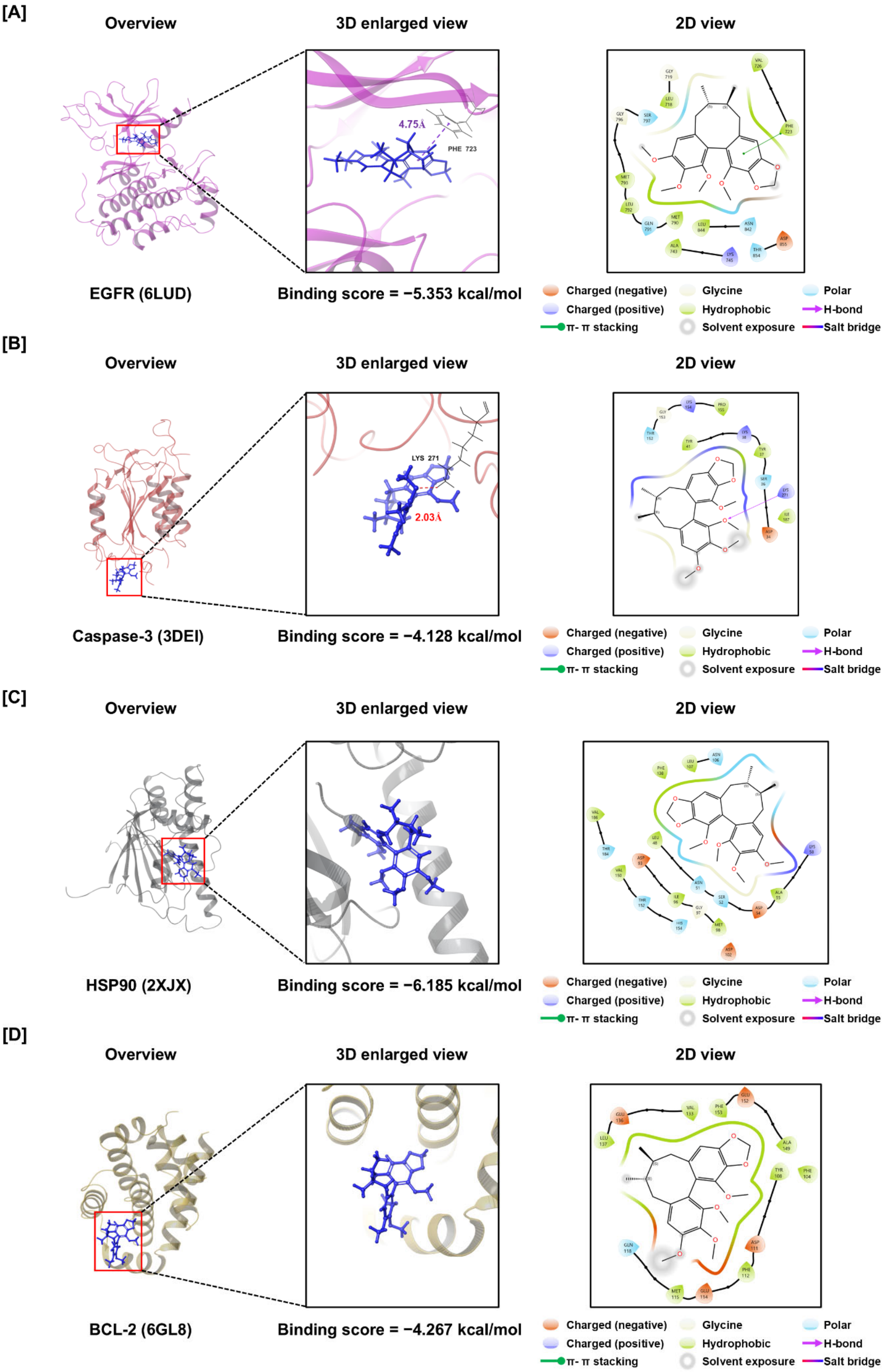

[A]
Overview
3D enlarged view
2D view
4.75Å
PHE 723
EGFR (6LUD)
Binding score = −5.353 kcal/mol
Charged (negative)
Charged (positive)
π- π stacking
Glycine
Hydrophobic
Solvent exposure
Polar
H-bond
Salt bridge
[B]
Overview
3D enlarged view
2D view
LYS 271
2.03Å
Caspase-3 (3DEI)
Binding score = −4.128 kcal/mol
Charged (negative)
Charged (positive)
π- π stacking
Glycine
Hydrophobic
Solvent exposure
Polar
H-bond
Salt bridge
[C]
Overview
3D enlarged view
2D view
HSP90 (2XJX)
Binding score = −6.185 kcal/mol
Charged (negative)
Charged (positive)
π- π stacking
Glycine
Hydrophobic
Solvent exposure
Polar
H-bond
Salt bridge
[D]
Overview
3D enlarged view
2D view
BCL-2 (6GL8)
Binding score = −4.267 kcal/mol
Charged (negative)
Charged (positive)
π- π stacking
Glycine
Hydrophobic
Solvent exposure
Polar
H-bond
Salt bridge

Figure S3. Molecular docking results depicting optimal conformation with lowest binding energy for schisandrin B and target proteins. The figure displays overview, enlarged 3D view, and 2D representation of the binding interactions between schisandrin B and the following target proteins: [A] EGFR (6LUD), [B] Caspase-3 (3DEI), [C] HSP90 (2XJX), and [D] BCL-2 (6GL8). The binding scores for schisandrin B with these targets were -5.353, -4.128, -6.185, and -4.267 kcal/mol, respectively. In the enlarged 3D views, interactions are illustrated with dashed lines: purple for π-π stacking, and red for hydrogen bonds.

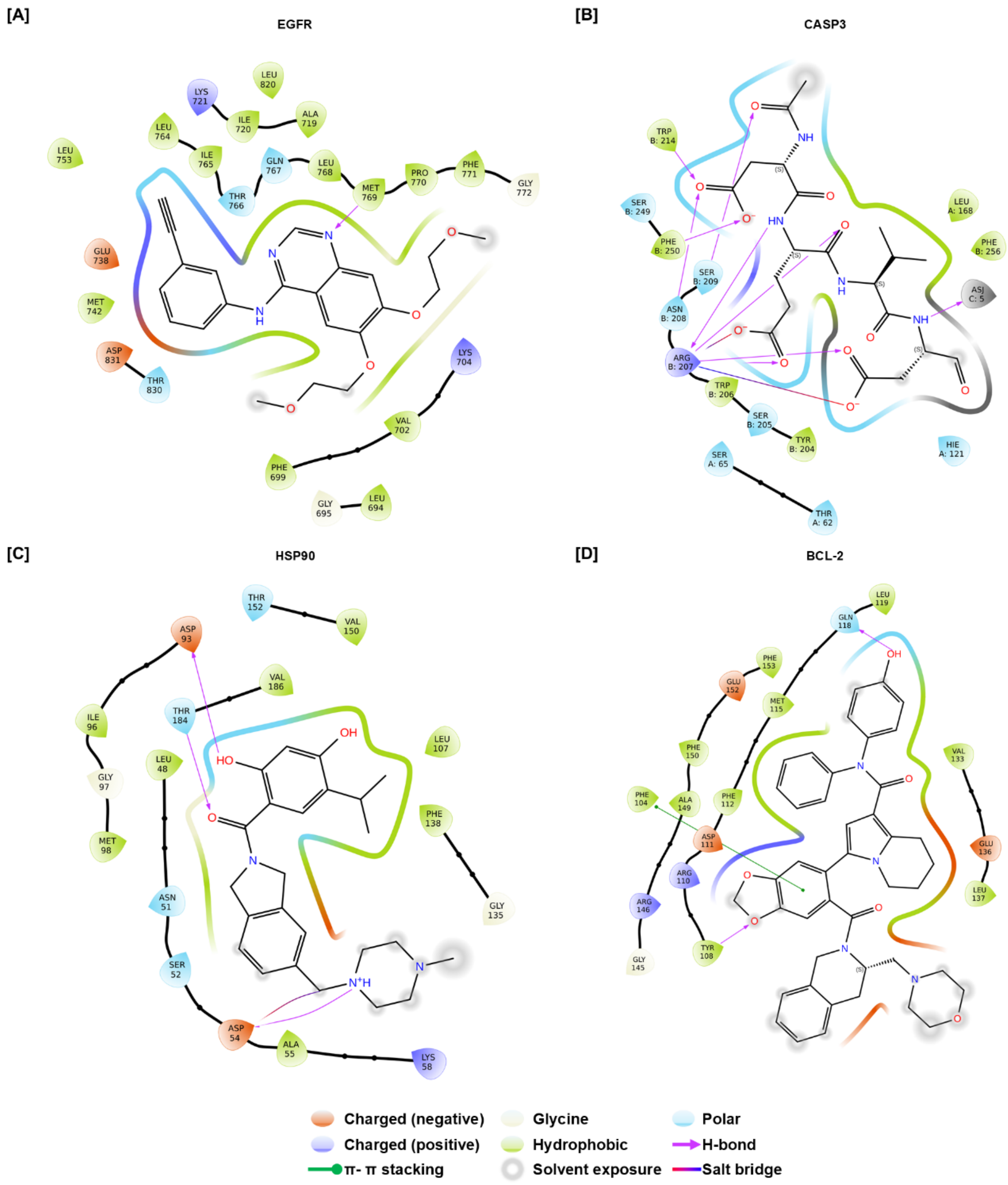

Figure S4. Predicted molecular docking interactions of reference inhibitors with candidate target proteins. Two-dimensional ligand-protein interaction diagrams showing the predicted binding interactions of [A] erlotinib with EGFR, [B] Ac-DEVD-CHO with CASP3, [C] onalespib with HSP90, and [D] S55746 with BCL-2. The diagrams illustrate the interacting amino acid residues and predicted intermolecular interactions.

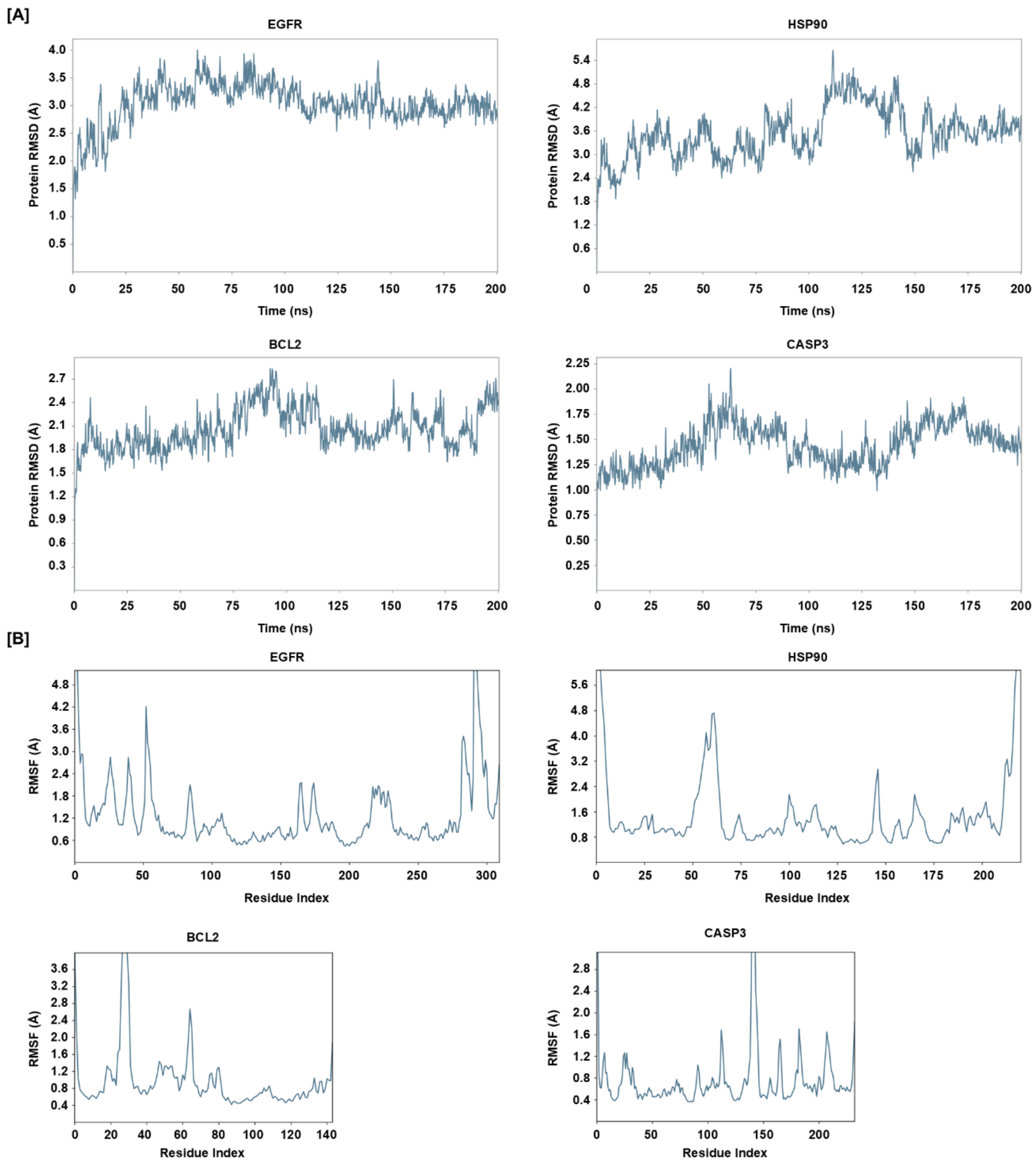

Figure S5. RMSD and RMSF analyses of the apo forms of EGFR, HSP90, BCL2, and CASP3 during 200 ns molecular dynamics simulations. [A] RMSD trajectories of the apo proteins over 200 ns. [B] RMSF profiles of the apo proteins by residue index. These apo-form simulations were conducted as ligand-free controls for comparison with the corresponding ligand-bound systems.

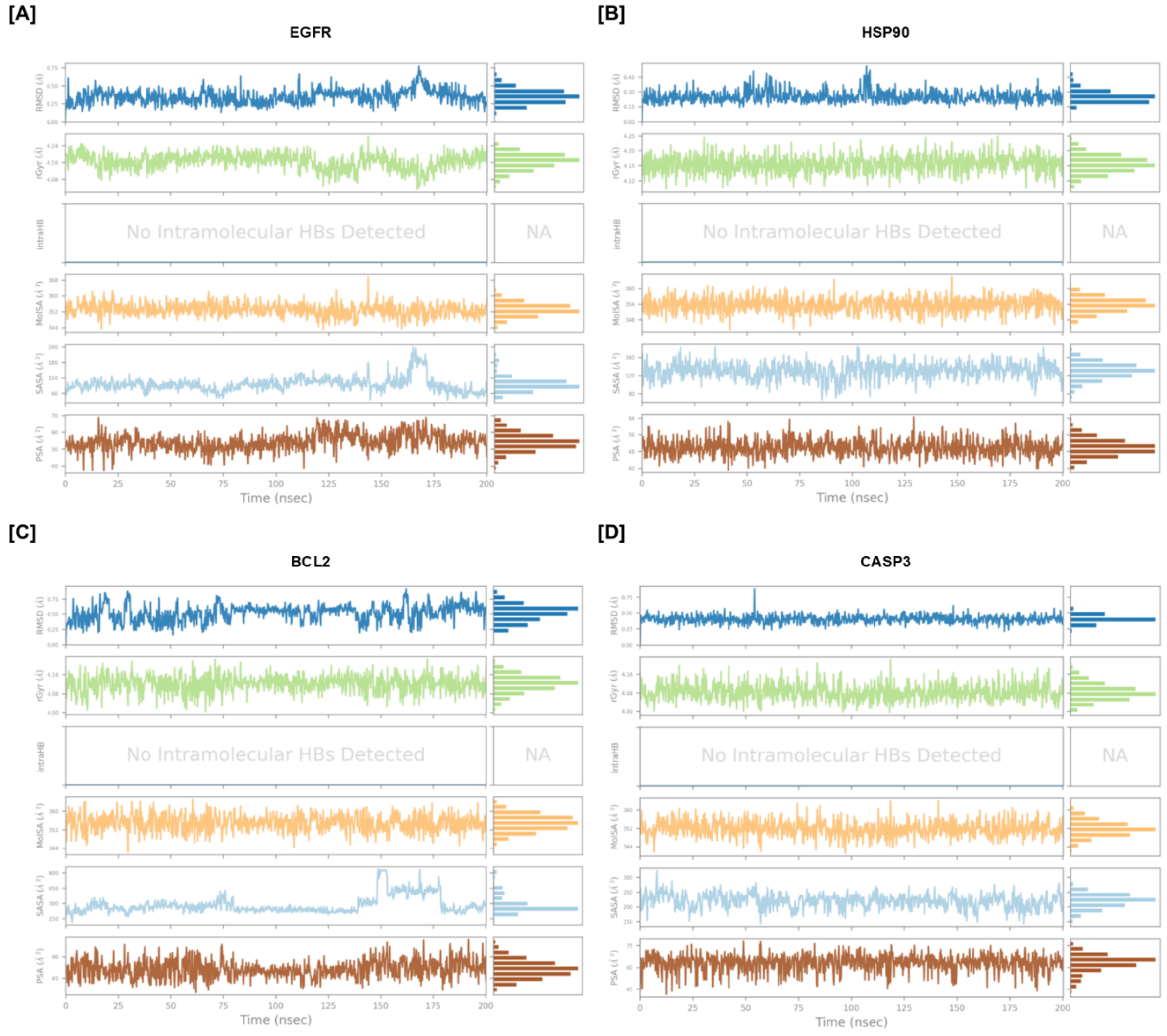

Figure S6. Ligand-specific structural properties of schisandrin B during 200 ns molecular dynamics simulations. Time-dependent ligand RMSD, radius of gyration (rGyr), intramolecular hydrogen bonds (intraHB), molecular surface area (MolSA), solvent-accessible surface area (SASA), and polar surface area (PSA) for Sch B in complexes with [A] EGFR, [B] HSP90, [C] BCL-2, and [D] CASP3. Histograms on the right summarize the distributions of the corresponding parameters throughout the simulations.

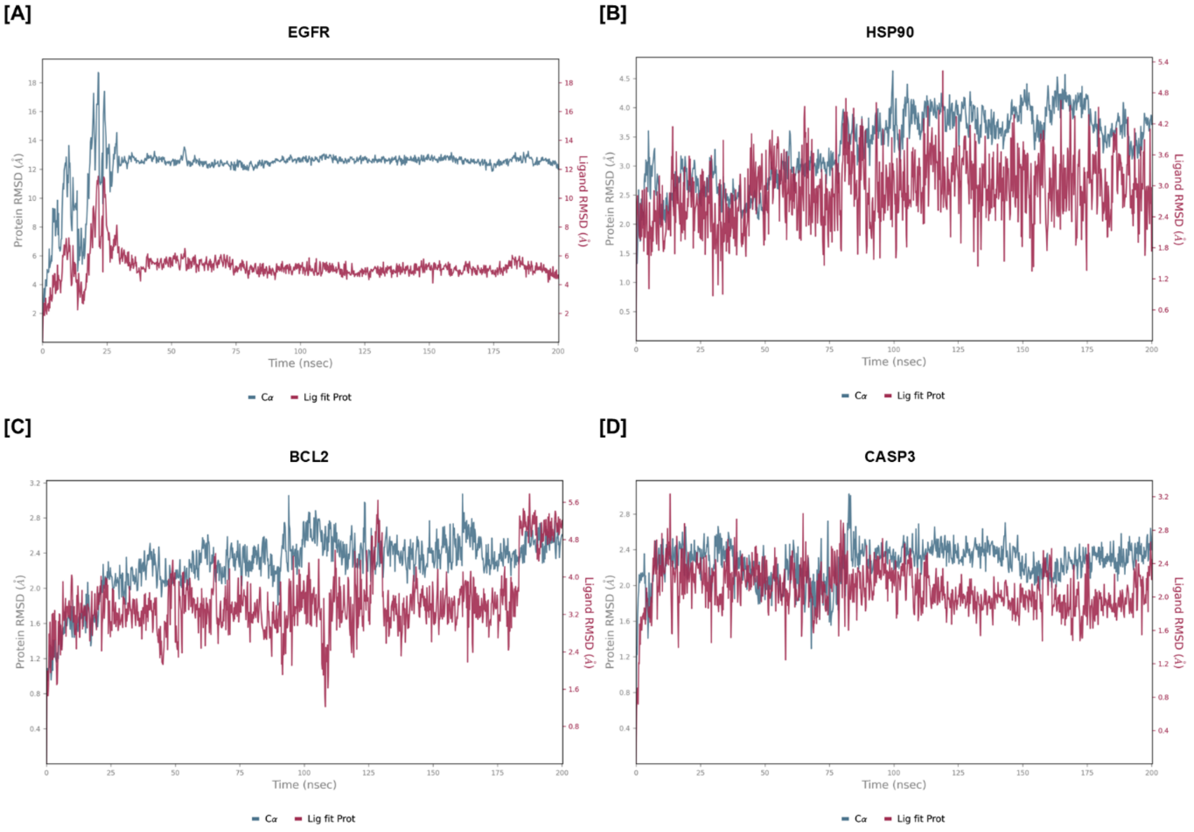

Figure S7. Protein and ligand RMSD analyses of reference inhibitor-bound complexes during 200 ns molecular dynamics simulations. Time-dependent protein Cα RMSD (blue) and protein-aligned ligand RMSD (red) for [A] EGFR-erlotinib, [B] HSP90-onalespib, [C] BCL-2-S55746, and [D] CASP3-Ac-DEVD-CHO complexes. The trajectories illustrate conformational deviations of the proteins and their corresponding reference inhibitors throughout the simulations.

Target protein	Reference inhibitor	Docking score (kcal/mol)	MM-GBSA (kcal/mol)
EGFR	Erlotinib	-8.727	-60.28
CASP3	Ac-DEVD-CHO	-5.288	-75.2
HSP90	Onalespib	-9.095	-68.07
BCL-2	S55746	-7.807	-58.51

Table S1. Comparative molecular docking results of schisandrin B and representative reference inhibitors with candidate target proteins.

Protein	PDB ID	UniProt ID	Mutation / modification	Resolution (Å)	Release date
EGFR	6LUD	P00533	T790M, C797S, L858R, E865A, E866A, K867A	2.05	2020-10-07
CASP3	3DEI	P42574	No sequence mutations; oxidized catalytic cysteine	2.8	2008-09-02
BCL-2	6GL8	P10415	L95K, Q99E; engineered construct	1.4	2018-11-07
HSP90	2XJX	P07900	No sequence mutations	1.66	2010-08-11

Table S2. Structural information of proteins selected for molecular docking. All protein structures were derived from *Homo sapiens* and determined by X-ray diffraction. Structural information was obtained from the RCSB Protein Data Bank.
